# From NMR to AI: Fusing ^1^H and ^13^C Representations for Enhanced QSPR Modeling

**DOI:** 10.1021/acs.jcim.5c01791

**Published:** 2025-09-17

**Authors:** Arkadiusz Leniak, Wojciech Pietruś, Rafał Kurczab

**Affiliations:** † Department of Medicinal Chemistry, 335807 Celon Pharma S.A., ul. Marymoncka 15, 05-152 Kazun Nowy, Poland; ‡ Department of Medicinal Chemistry, Maj Institute of Pharmacology, Polish Academy of Sciences, Smetna 12, 31-343 Krakow, Poland

## Abstract

The ability to predict log *D* directly from spectral patterns marks a conceptual shift in cheminformatics. In this work, we demonstrate that ^1^H and ^13^C NMR spectra, computationally generated from molecular structures and transformed into machine learning-compatible vectors, can approach and rival classical structure-based descriptors such as ECFP4 fingerprints in modeling the log *D* parameter. Through comprehensive benchmarking of nearly 70 models across seven algorithmic classes and three pH conditions, we show that concatenation of ^1^H and ^13^C NMR spectra offers the best trade-off between accuracy and efficiency. In the best case, a fused spectral CNN model achieved a root-mean-square error (RMSE) of 0.57 and a *Q* ^2^ of 0.76 using a 400-dimensional input vector—closely matching the ECFP4 benchmark (RMSE 0.56, *Q* ^2^ 0.78) despite being five times smaller. These findings challenge the assumption that descriptor richness must come at the cost of dimensional complexity. SHAP-based analysis revealed modality-specific patterns: ^13^C regions linked to aromatic and carbonyl carbons (110–170 ppm) increased predicted log *D*, while ^1^H signals associated with polar groups, including OH, NH, amides, and ethers (2–4.5 and ∼8 ppm), reduced it. This positions NMR-based vectors as both interpretable and scalable alternatives to conventional fingerprints. By releasing a standalone graphical prediction tool based on our models, we make this paradigm practically accessible for real-world applications. This study establishes *in silico-*generated NMR spectra as valid and powerful descriptors in predictive modeling, paving the way for spectrum-driven approaches to drug discovery and property prediction.

## Graphical abstract

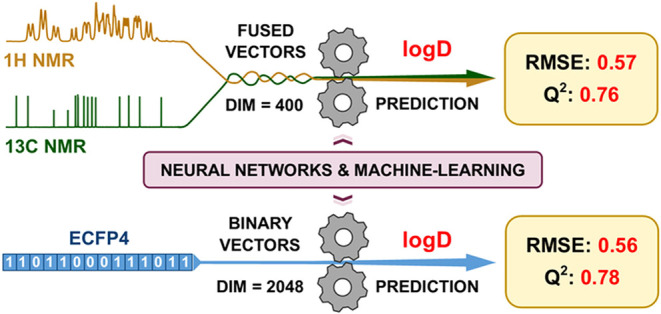

## Introduction

Predictive modeling of molecular physicochemical properties has long relied on two-dimensional (2D) structural descriptors, most notably extended-connectivity fingerprints (ECFP, 2048 bits),^1,2^ which encode substructure presence via high-dimensional binary vectors. While these fingerprints can deliver respectable performance, they impose substantial compute and storage overhead and frequently require GPU-accelerated hardware to support large-scale hyperparameter sweeps.^3,4^ Even the latest graph-based and quantum chemistry-informed models benchmarked in MoleculeNet seldom surpass *R* ^2^ ≈ 0.80 for key end points without multi-GPU training or explicit QM descriptors.^5–7^ Moreover, plateauing returns in model accuracy suggest that purely topological representations lack the electronic and conformational nuances intrinsic to real-world compound behavior. Venkatraman et al. demonstrated that these conventional descriptors often fail to distinguish between active and inactive compounds in large-scale data sets, thereby reducing their utility in comprehensive screening campaigns.^8^ Similarly, Boldini et al. underscored the restricted capacity of traditional fingerprints to fully represent the rich structural diversity found in natural products, which poses significant challenges for accurate predictive modeling.^9^ Gao et al. emphasized that while 2D fingerprints remain prevalent in drug discovery pipelines, their reliance on static, expert-defined heuristics and simplified connectivity patterns makes them insufficient for capturing the dynamic three-dimensional (3D) and physicochemical properties that govern molecular recognition.^10^ Furthermore, van Tilborg et al. highlighted the inability of such representations to account for subtle yet critical structural variations—known as activity cliffs—that can dramatically alter biological activity, thereby compromising the generalizability and interpretability of predictive models.^11^ To break through this ceiling, alternative representations that embed three-dimensional and electronic-environment information are required—ideally in a form amenable to efficient featurization and downstream machine learning (ML).^12^

Nuclear magnetic resonance (NMR) spectra intrinsically capture chemical–environment information, including electron distribution, conformation, and intermolecular interactions, all of which are critical for accurate property prediction yet are unavailable in standard fingerprints. In our initial study,^13^ experimental ^1^H NMR spectra were transformed into bucketed vectors and shown to predict chromatographic log *D* with accuracy comparable to 2D descriptors. Subsequently, fully computational workflows—employing NMRshiftDB2 ^14,15^ and JEOL JASON predictors^16^—demonstrated that simulated ^1^H spectra can substitute experimental data without loss of model quality while alleviating bottlenecks of sample preparation and spectral acquisition.^17^ This advancement has enabled the *in silico* generation of NMR spectra and high-throughput property prediction across vast virtual libraries, decoupling model development from physical compound availability.

While early quantitative structure–activity relationship (QSAR) efforts focused on small steroidal cohorts, several studies over the past decade have demonstrated that ^13^C NMR data can serve as effective electronic descriptors in supervised property prediction workflows. For example, simulated ^13^C NMR shifts were encoded into self-organizing (Kohonen) maps and combined with topological indices to predict the antioxidant activity of flavonoids, achieving *Q* ^2^ ≈ 0.84.^18^ As early as 1994, bucketed ^13^C NMR bins were incorporated into PLS regressions to predict acute-toxicity end points for aquatic organisms, achieving *R* ^2^ ≈ 0.72,^19^ and in 2002, similar ^13^C-only QSAR models matched classical QSAR performance for aromatase inhibitors (*R* ^2^ ≈ 0.78).^20^ However, in the two decades following these pioneering studies, reports of ^13^C NMR-based quantitative structure–property relationship (QSPR) or QSAR models have been scarce, with most subsequent work reverting to classical fingerprints or focusing solely on ^1^H data.^21,22^ Despite decades of research, no supervised machine learning framework with rigorous hyperparameter optimization has been devised to directly ingest predicted ^13^C NMR spectra for QSPR end points, and models achieving robust, generalizable performance remain elusive.

However, all of our prior models were built with default hyperparameters and limited to ^1^H NMR spectra alone. The potential of ^13^C NMR data remains unexplored, and the impact of rigorous algorithmic tuning on NMR-based representations is unknown. In the present work, fusion strategies are introduced to combine predicted ^1^H and ^13^C spectra into compact vectors, yielding feature sets 5-fold smaller than ECFP4, while comprehensive hyperparameter optimization ensures maximal performance. As a case study, log *D* prediction models trained on single-modal and fused spectral inputs are benchmarked against the classical ECFP4 fingerprint, with outcomes intended to validate low-dimensional NMR vectors as both efficient and interpretable alternatives for property prediction, thereby opening a new paradigm in spectral-driven QSPR.

## Materials and Methods

### Compound Data Set

Two separate data sets were utilized in this study: the old data set—used in earlier publications,^13,17^ and a newly curated data set—developed specifically for the current research.

The new data set includes all compounds from the previous data set and has more than doubled in size through the addition of new molecules sourced from the internal Celon Pharma database (Table 1), representing various stages of the drug design and development process. Canonical SMILES strings were converted to 2048-bit Morgan fingerprints (radius = 2) with RDKit; pairwise similarities were quantified as Tanimoto coefficients, transformed to distances (*d* = 1 – *T*_c_), and submitted to agglomerative hierarchical clustering with an average linkage. The dendrogram was truncated at *d* = 0.30, equivalent to *T*_c_ = 0.70.

**Table 1:** Number of Compounds with CHI log *D* Values Used in Modeling at Each pH Level

data set	pH 2.6	pH 7.4	pH 10.5
old data set	596	589	595
new data set	1262	1276	1223

Compound lipophilicity was characterized exclusively using the CHI log *D* parameter, a chromatographically derived descriptor inferred from the analyte retention time as detailed by Mach et al. and described in a previous study.^23^

### Preparation of Spectral and Molecular Representations

To automate the generation of machine learning input data based on both simulated spectral and structural molecular representations, the software platform Demiurge was implemented in Python. Demiurge processes input data in the form of csv files containing compound structures encoded as SMILES strings along with the associated target values (in this work, CHI log *D*) and produces ready-to-use feature matrices tailored for both classical ML algorithms and neural networks. The software supports three types of molecular representations: predicted ^1^H NMR spectra, predicted ^13^C NMR spectra, and ECFP4 ^1^ molecular fingerprints. For the prediction of ^1^H and ^13^C NMR spectra, Demiurge employs a Java-based standalone predictor built upon the NMRshiftDB2 database.^14,15,24^ The output consists of lists of predicted chemical shift values corresponding to individual H or C atoms in the molecule. Since the data consist of shift lists rather than continuous spectra, a bucketing approach was used to transform them into fixed-length feature vectors for machine learning. In this study, the ^1^H NMR region (−1 to 16 ppm) and the ^13^C NMR region (−10 to 230 ppm) were each divided into 200 equal-width buckets using a previously developed approach.^13^ This process ensures that all molecular spectra are transformed into vectors of identical dimensionality regardless of the number or type of atoms present in the molecule. As an alternative structural representation, Demiurge also supports the generation of ECFP4 using RDKit’s Morgan fingerprinting algorithm with a radius of 2 (2048 bits). The architecture of Demiurge is fully modular and extensible, allowing for straightforward adaptation to other end points such as log *P*, TPSA, or aqueous solubility (log *S*), as well as to alternative spectral formats. More details are available in the Supporting Information or on the GitHub repository page.

### Machine Learning Models and Hyperparameter Optimization

All workflows were managed via MLflow to track experiments, model versions, and log hyperparameters.^25^

Before model training, all machine learning algorithms and neural network architectures underwent comprehensive hyperparameter optimization using the Optuna framework.^26^ Optuna is an advanced, heuristic-based Bayesian optimization tool that enables efficient and adaptive exploration of high-dimensional parameter spaces. For each model class, Optuna additionally provided parameter importance weights, estimating the relative contribution of individual hyperparameters to the final model performance. These weights were used in the subsequent analysis of the model behavior and architecture sensitivity.

Hyperparameter tuning was applied not only to classical machine learning models but also to the architectural components of neural networks. Detailed information on the relative importance of individual hyperparameters is provided in the Supporting Information (Tables S7–S11). The complete list of all hyperparameters subjected to optimization, along with their respective search ranges for each algorithm and neural architecture, is documented in Table S12.

To enable benchmarking against prior studies, the machine learning models evaluated in this work included support vector regression (SVR)^27^ using the GPU-accelerated implementation from the cuML library (NVIDIA RAPIDS) with RBF kernel and extreme gradient boosting (XGBoost) using the native xgboost Python package.^28^ The RBF kernel was applied in SVR due to its ability to model nonlinear relationships by implicitly mapping spectral vectors into a high-dimensional feature space using the kernel trick, enabling the model to learn local dependencies without explicit transformation.^29^ XGBoost was selected over classical gradient boosting due to its native GPU support and advanced algorithmic enhancements, including regularization mechanisms and second-order gradient optimization, which collectively yield superior model accuracy and training efficiency. These features proved essential, given our high-throughput tuning approach relying on thousands of model evaluations. Indeed, comparative studies affirm that XGBoost offers both faster convergence and better generalization compared to conventional implementations.^28,30^ In addition, two main classes of deep neural networks were implemented and evaluated: multilayer perceptrons (MLPs) and convolutional neural networks (CNNs). All neural networks were implemented using the PyTorch framework,^31^ which provided the necessary flexibility for architectural design and optimization across all configurations.

All trained models were evaluated using 10-fold cross-validation (10CV), allowing robust assessment of model generalization performance across diverse data splits. For each model type, a fixed number of Optuna trials was used to ensure reproducibility: 2000 trials for XGBoost and neural networks and 5000 trials for SVR, which required significantly less computational time per evaluation.

All codes, including data preprocessing pipelines, model training scripts, optimization routines, and configuration files, are publicly available, along with documentation in a dedicated GitHub repository.

### Generation of Hybrid Spectral Representations

Several strategies were implemented to fuse predicted ^1^H and ^13^C NMR spectra into hybrid representations (Figure 1).

**Figure 1:**
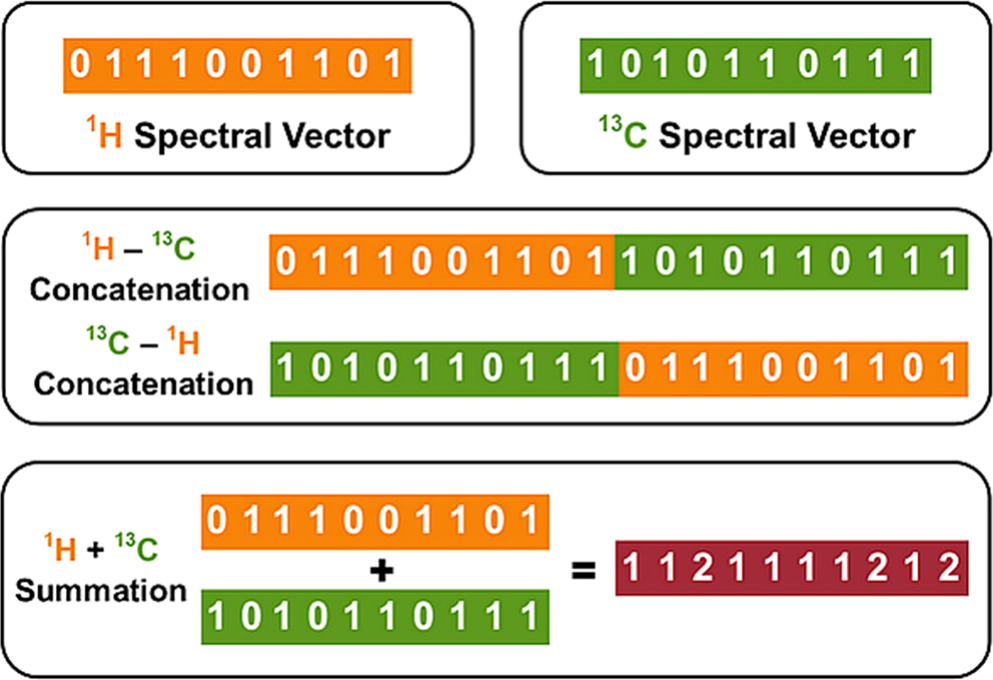
Schematic overview of hybrid spectral representation strategies. Individual ^1^H and ^13^C NMR spectral vectors (200 dimensions each) were combined by direct concatenation (resulting in 400 dimensions) or element-wise summation (200 dimensions).

In vector concatenation, the two 200-dimensional spectral vectors corresponding to ^1^H and ^13^C NMR were joined sequentially to form a single 400-dimensional vector. Two concatenation variants were prepared: one with the ^1^H vector preceding the ^13^C vector and in reversed order. In the element-wise summation approach, the values of the corresponding elements from the ^1^H and ^13^C vectors were added together. The resulting vector retained the original dimensionality of 200 elements and was used as a compact fused representation. This operation carries no strict physicochemical interpretation as the spectral domains of ^1^H and ^13^C NMR differ fundamentally. It was introduced purely as a technical means to achieve additive compression of two spectral vectors into a fixed-length representation.

Three dual-input architectures for joint ^1^H|^13^C fusion were implemented in PyTorch (Figure 2). All input vectors were standardized prior to model construction, as described earlier.

**Figure 2:**
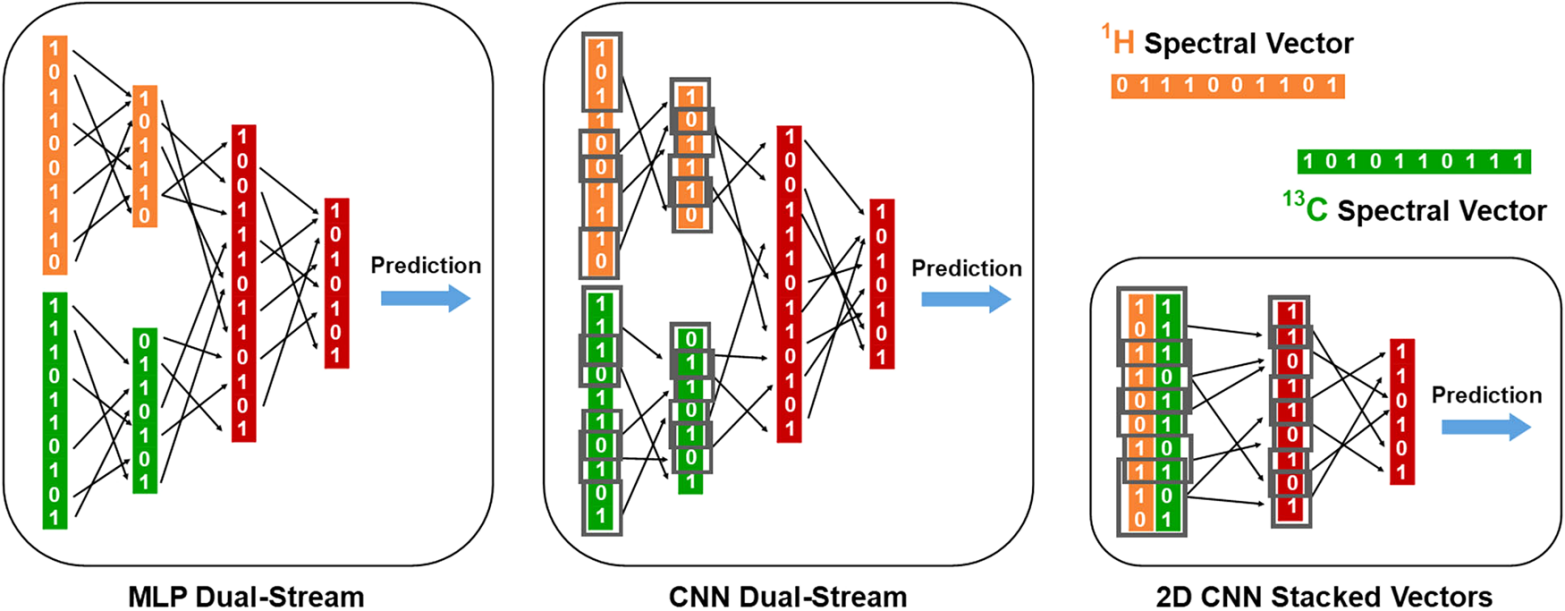
Overview of hybrid neural architectures integrating ^1^H and ^13^C NMR spectral representations. MLP dual-stream, CNN dual-stream, and 2D CNN stacked vectors are shown. Orange and green blocks represent the processing of ^1^H and ^13^C NMR vectors, respectively; red blocks denote shared layers producing the final prediction. Gray rectangles indicate convolutional filters applied in CNN-based models.

The MLP dual-stream architecture processes ^1^H and ^13^C NMR spectra through separate fully connected branches, optionally linked by a cross-attention mechanism that enables each modality to attend to informative features in the other. The CNN dual-stream variant replaces dense layers with one-dimensional (1D) convolutional blocks tailored for spectral data, capturing local patterns and optionally leveraging the same bidirectional attention module for cross-modal integration. In contrast, the CNN 2D stacked-spectral model combines both spectra into a two-channel input and applies initial 2D convolutions to capture early interspectral interactions, followed by 1D convolutions along the chemical shift axis to refine joint feature representations.

### Assessment of Predictive Model’s Performance

The predictive performance of all trained models was evaluated based on four criteria: RMSE, *Q* ^2^, *R* ^2^_train, and the applicability domain (AD). The evaluation strategy adopted in this study aligns with widely accepted OECD guidelines,^32^ as well as best practices recommended by Tropsha^33^ and Gramatica.^34^

Root-mean-square error (RMSE) quantifies the average magnitude of prediction errors and is expressed in the same units as the modeled end point. In the context of lipophilicity modeling, RMSE values below 1.0 are typically considered acceptable, while values around or below 0.6 are increasingly regarded as characteristic of high-quality models. For instance, in the SAMPL7 blind prediction challenge, none of the submitted log *P* models achieved RMSE below 0.6, with the best entries ranging from 0.63 to 0.80—underlining the difficulty of reaching this threshold in real-world settings.^35^ Similarly, Plante and Werner reported RMSE ≈ 0.60 for their JP log *P* model and explicitly noted that such performance was close to the experimental uncertainty and significantly better than most established methods.^36^ More recently, a model predicting log *D* at pH 7.4 reported RMSE 0.50, outperforming traditional descriptors and transfer learning baselines.^37^ These reports position RMSE ≈ 0.60 as a competitive and realistic benchmark for models targeting log *P* or log *D*, especially when trained on diverse molecular sets or alternative representations.

The coefficient of determination obtained via cross-validation (*Q* ^2^) reflects the model’s predictive power and generalizability by quantifying the proportion of variance explained on unseen subsets of data. *Q* ^2^ values above 0.5 are generally considered the minimum threshold for predictive usefulness in QSPR modeling. However, robust and truly predictive models typically exceed *Q* ^2^ 0.7, indicating both stable generalization and reliable performance across the chemical space. This stricter threshold has been emphasized in several critical assessments of model validation strategies.^34,38^

The coefficient of determination calculated for the training set (*R* ^2^_train) specifically measures how accurately the trained model fits the data used directly in the training process. While high *R* ^2^_train values alone do not guarantee good predictive performance on unseen data, they are critical in identifying potential overfitting. Following best practices in QSAR validation, *R* ^2^_train values above 0.8 are generally acceptable, while values exceeding 0.9 suggest a strong fit to the training data.^33,34^

The applicability domain (AD) analysis assesses the reliability of the model’s predictions by identifying molecules whose chemical structure or property space lies significantly outside the region covered by the training set. Models with fewer than 15% of data points flagged as outliers (outside the AD) are considered acceptable, whereas outlier percentages below 10% suggest good reliability.^32,34^ According to Gramatica,^34^ models with outlier rates below 5% demonstrate excellent applicability domain coverage.

To assess the applicability domain (AD) of the developed models, Williams plots were generated for selected representative models.^34^ Plots show standardized residuals versus leverage for each training instance. The leverage threshold was set as $h^{*}=\frac{3p}{n}$ , where *p* is the featuring count and *n* is the training size. Compounds with leverage above the h* threshold or standardized residuals outside the ± 3 range were considered influential or poorly predicted outliers, respectively. The resulting plots and analyses provide insight into the robustness, generalizability, and potential limitations of each model concerning the chemical diversity of the data set.

Tables containing detailed metrics (RMSE, *Q* ^2^, *R* ^2^_train) for each evaluated model are comprehensively documented in the Supporting Information accompanying this work.

## Results

### Data Set Analysis

The consolidated library encompasses 1290 aromatic compounds spanning 176–2790 Da and exhibiting CHI log *D* envelopes of −1.36 to 3.40 (pH 2.6), 0.22–5.08 (pH 7.4), and −0.57 to 5.86 (pH 10.5) (Table S1). Morgan fingerprint partitioning with a Tanimoto cutoff of 0.75—widely adopted as the operational boundary for scaffold-level similarity in cheminformatics workflows^39,40^ —yielded 43 structurally coherent clusters ranging from singletons (12 clusters, 28%) to a high-density trifluoromethyl-phenylpyrimidine cluster (317 molecules) (Figure 3). The five largest disclosed clusters, together accounting for 73% of all molecules, demonstrate substantial intracluster diversity: the aforementioned phenylpyrimidines (183–583 Da; log *D* ranges −1.36 to 3.36/0.22–4.02/– 0.34 to 4.66, respectively, at pH 2.6/7.4/10.5), difluorophenyl piperazines (248–504 Da; – 1.22 to 4.12/0.31–4.45/– 0.45 to 5.82), indole-carboxamides (308–620 Da; – 0.01 to 4.27/1.73–5.04/1.72–5.73), sulfonamides (347–604 Da; −0.78 to 3.19/0.50–4.34/–0.04 to 5.73) and benzoxazole amides (320–569 Da; −0.37 to 3.95/0.78–5.08/0.84–5.82).

**Figure 3:**
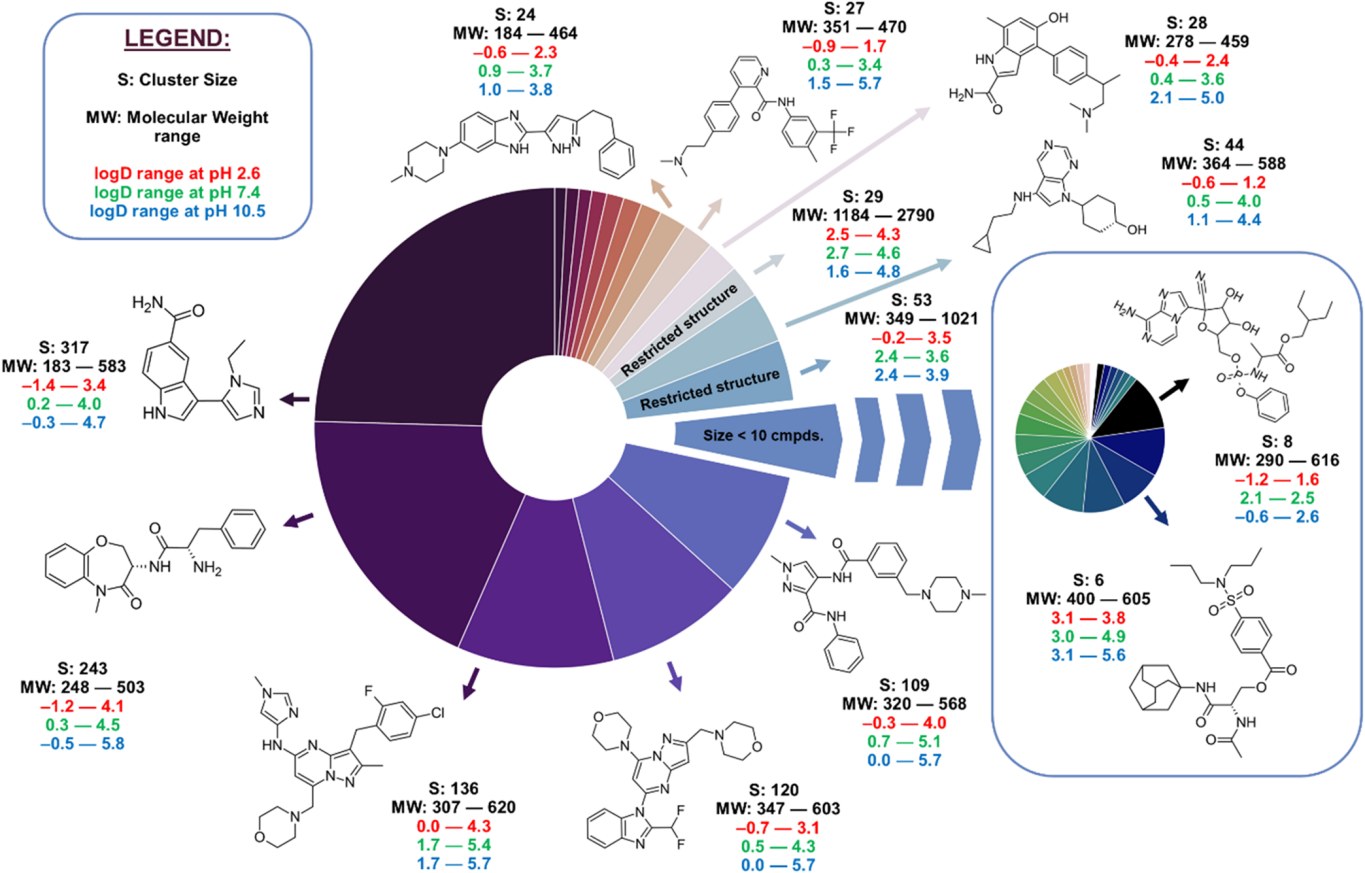
Cluster-size distribution of the 1290 compound library, together with representative chemotypes and their physicochemical windows. Peripheral callouts give chemotype representatives with cluster size (S), intracluster MW range, and CHI log *D* ranges at pH 2.6 (red), 7.4 (green), and 10.5 (blue), while restricted series are flagged. The inset pie zooms into the aggregate slice representing clusters with <10 molecules.

The difluorophenyl piperazine cluster exhibits the broadest log *D* dispersion (6.27 units across the three pH conditions), whereas a macrocyclic cluster (29 molecules) shows the widest MW spread (1184–2790 Da). Three restricted clusters, each comprising large, high-lipophilicity molecules (*M*_W_ ≈ 1.2 – 2.8 kDa; log *D*_2.6_ ≈ 2.5 – 4.3), are held back from public disclosure pending patent finalization. Collectively, the cluster architecture delivers a scaffold-balanced landscape suitable for robust model development, rigorous applicability domain assessment, and high-variance external validation.

### ^1^H Spectral Models’ Performance

In this study, we re-evaluated model performance by training on the previously used data set^17^ with the addition of hyperparameter optimization and subsequently on a new, expanded data set under the same optimized settings. In the original, nonoptimized configuration, gradient boosting achieved RMSE values of 0.87, 0.76, and 0.94 at pH 2.6, 7.4, and 10.5, respectively, with *Q* ^2^ values ranging from 0.43 to 0.54. SVR performed considerably worse under these conditions, especially at 2.6 and 10.5 pH, yielding RMSE values of 1.03 and 1.07, with corresponding *Q* ^2^ values of 0.36 and 0.25 (Figure 4). Training performance (*R* ^2^_train) also remained moderate across models, with XGBoost ranging from 0.85 to 0.88 and SVR dropping as low as 0.51 (Figure S1).

**Figure 4:**
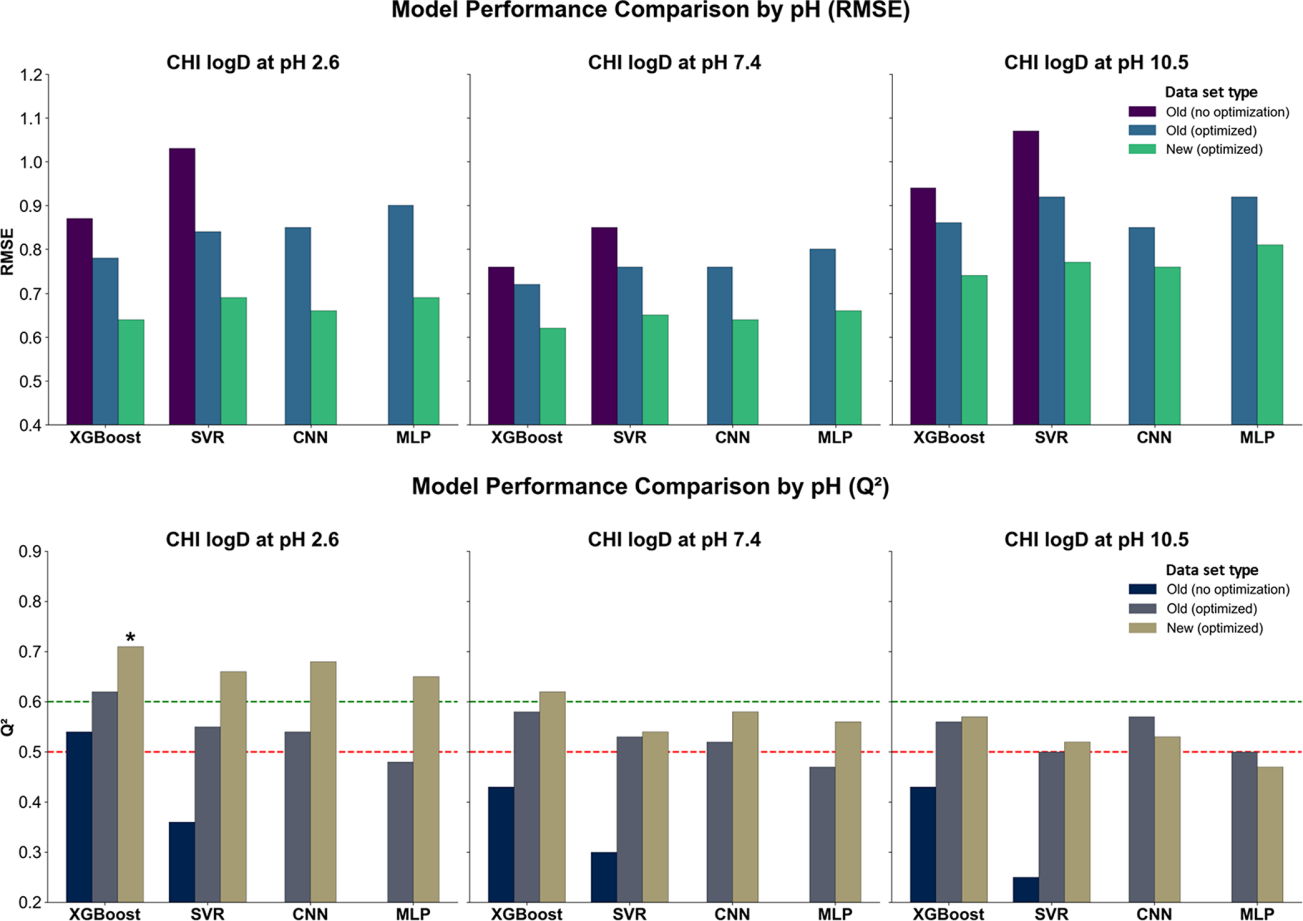
RMSE and *Q* ^2^ of all models trained using ^1^H NMR-based spectral representations at pH 2.6, 7.4, and 10.5. Bars are grouped by the training data set version (old, nonoptimized; old, optimized; new, optimized) and ML model. Red and green lines mark *Q* ^2^ thresholds of 0.5 (minimal predictive value) and 0.6 (acceptable model), respectively; *Q* ^2^ ≥ 0.7 indicates a good model. Black asterisks above bars denote models exceeding this threshold.

Following optimization, all models demonstrated substantial improvements. On the old data set, XGBoost achieved RMSE values of 0.78, 0.72, and 0.86 (*Q* ^2^ at 0.62, 0.58, and 0.56), and SVR returned RMSE values of 0.84, 0.76, and 0.92 (*Q* ^2^ at 0.55, 0.53, and 0.50). CNN showed competitive performance, particularly at pH 7.4 and 10.5, where it achieved RMSE values of 0.76 and 0.85, with *Q* ^2^ values of 0.52 and 0.57, respectively. MLP remained slightly less effective overall, with RMSE values of 0.90, 0.80, and 0.92 across the three pH levels and *Q* ^2^ ranging from 0.48 to 0.50. Despite this, all optimized models reached *R* ^2^_train values above 0.83, with most exceeding 0.90, confirming robust convergence during training.

The best overall performance, however, was obtained using the new, optimized data set. At pH 2.6, XGBoost yielded the lowest RMSE (0.64) and highest *Q* ^2^ (0.71), followed by CNN and MLP (0.66/0.68 and 0.69/0.65, respectively). SVR was slightly behind, with RMSE at 0.69 and *Q* ^2^ at 0.66. At pH 7.4, XGBoost again led with RMSE 0.62 and *Q* ^2^ at 0.62, while CNN and MLP followed closely (0.64/0.58 and 0.66/0.56). SVR returned an RMSE of 0.65 and *Q* ^2^ at 0.54. Under basic conditions (pH 10.5), the differences between the models were less pronounced. XGBoost, CNN, and MLP reached RMSE values of 0.74, 0.76, and 0.81, with *Q* ^2^ ranging from 0.47 to 0.57. SVR once again showed slightly weaker performance (0.77/0.52). Nonetheless, all models trained on the new data set demonstrated high *R* ^2^_train values, exceeding 0.85 for SVR and reaching up to 0.98 for XGBoost, further supporting the benefits of both data augmentation and hyperparameter tuning in improving predictive accuracy.

### ^13^C Spectral Models’ Performance

To parallel the analysis performed on the ^1^H NMR data, we constructed a comprehensive set of models based on simulated ^13^C NMR spectra. Following the same logic, we compared models trained on the old nonoptimized data set, those trained on the same data with full hyperparameter tuning, and finally, models built on the expanded data set under optimization (Figure 5).

**Figure 5:**
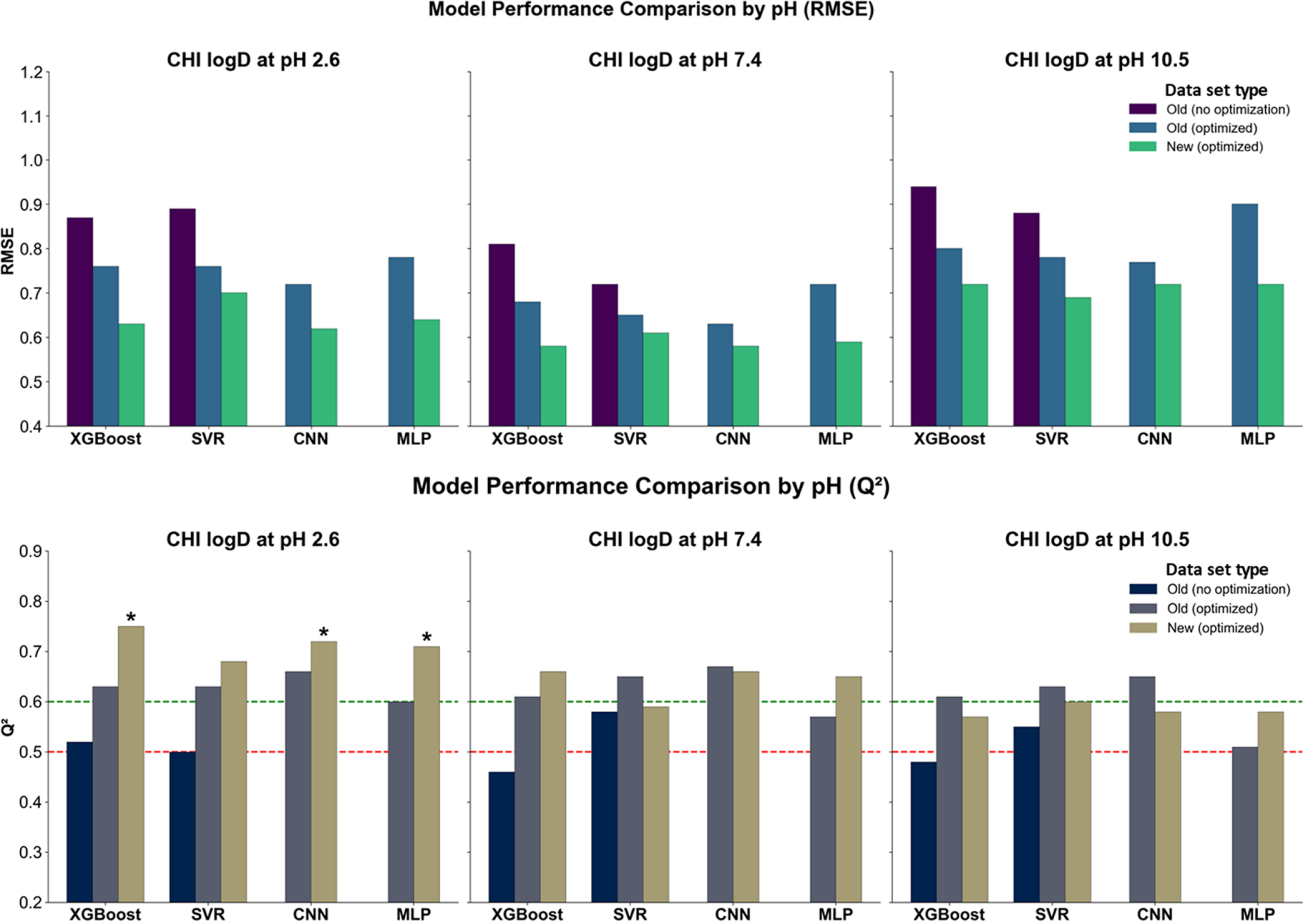
RMSE and *Q* ^2^ of all models trained using ^13^C NMR-based spectral representations at pH 2.6, 7.4, and 10.5. Red and green lines mark *Q* ^2^ thresholds of 0.5 (minimal predictive value) and 0.6 (acceptable model), respectively; *Q* ^2^ ≥ 0.7 indicates a good model. Black asterisks above bars denote models exceeding this threshold.

On the nonoptimized data set, SVR and XGBoost exhibited comparable performance across pH conditions, with RMSE values ranging from 0.72 to 0.94 and *Q* ^2^ between 0.46 and 0.58. At pH 7.4, SVR achieved the lowest RMSE of 0.72 (*Q* ^2^ at 0.58), while XGBoost showed a stronger performance at pH 2.6 (RMSE at 0.87 and *Q* ^2^ at 0.52). The predictive quality remained modest overall, and training fits were moderate with *R* ^2^_train values consistently between 0.74 and 0.79 (Figure S1).

Optimization led to clear improvements in both the RMSE and *Q* ^2^ across all algorithms. At pH 2.6, CNN outperformed the other models (RMSE 0.72/*Q* ^2^ 0.66), followed by XGBoost and SVR (both 0.76/0.63), while MLP trailed slightly (0.78/0.60). Similar trends were observed at pH 7.4, where CNN again led with RMSE at 0.63 and *Q* ^2^ at 0.67, with SVR and XGBoost performing similarly (0.65/0.65 and 0.68/0.61, respectively). At pH 10.5, CNN maintained its advantage (0.77/0.65) while SVR and XGBoost remained close (0.78/0.63 and 0.80/0.61, respectively). MLP consistently underperformed under basic conditions, with *Q* ^2^ dropping to 0.51. All optimized models trained on the old data set exhibited high *R* ^2^_train values, exceeding 0.94 and reaching up to 0.99 in the case of CNN.

Training on the expanded data set brought further performance gains, particularly at pH 2.6 and 7.4. For both pH values, CNN and XGBoost achieved the best results, with RMSE values of 0.62–0.63 and *Q* ^2^ in the range of 0.72–0.75. MLP also improved considerably (0.64/0.71 at pH 2.6 and 0.59/0.65 at pH 7.4), while SVR, although slightly behind, remained competitive. At pH 10.5, the performance declined slightly across all models. RMSE values converged to approximately 0.72 for CNN, XGBoost, and MLP, with corresponding *Q* ^2^ values of 0.58, 0.57, and 0.58, respectively. SVR remained marginally less accurate, with an RMSE of 0.69 and *Q* ^2^ at 0.60. Despite these differences, all optimized models trained on the expanded data set displayed excellent training convergence, with *R* ^2^_train values exceeding 0.93 in all cases.

Taken together, CNN emerged as the most consistently robust model across data sets and pH values, particularly under optimized conditions. XGBoost and SVR also demonstrated stable and high performance. The effect of hyperparameter optimization was pronounced, improving both the prediction quality and model consistency. Furthermore, expanding the training set significantly enhanced performance across architectures, notably increasing *Q* ^2^ and reducing RMSE variance, especially in the acidic and neutral pH range.

### Performance of Models Based on Fused ^1^H/^13^C Spectral Representations

To assess the predictive advantages of combining ^1^H and ^13^C NMR inputs, we constructed a series of models using concatenated spectral vectors in three configurations: ^1^H|^13^C, ^13^C|^1^H, and an element-wise summation (^1^H + ^13^C) (Figure 1). At pH 2.6, the best results were obtained for the ^1^H|^13^C setup, where CNN and XGBoost achieved *Q* ^2^ at 0.76, with RMSE values of 0.57 and 0.59, respectively (Figure 6). MLP and SVR yielded slightly inferior predictions with *Q* ^2^ values of 0.73 and 0.72 and RMSE values of 0.61 and 0.63, respectively. The reversed order (^13^C|^1^H) produced virtually identical metrics, differing by less than 0.01 in both RMSE and *Q* ^2^ (Figure 1).

**Figure 6:**
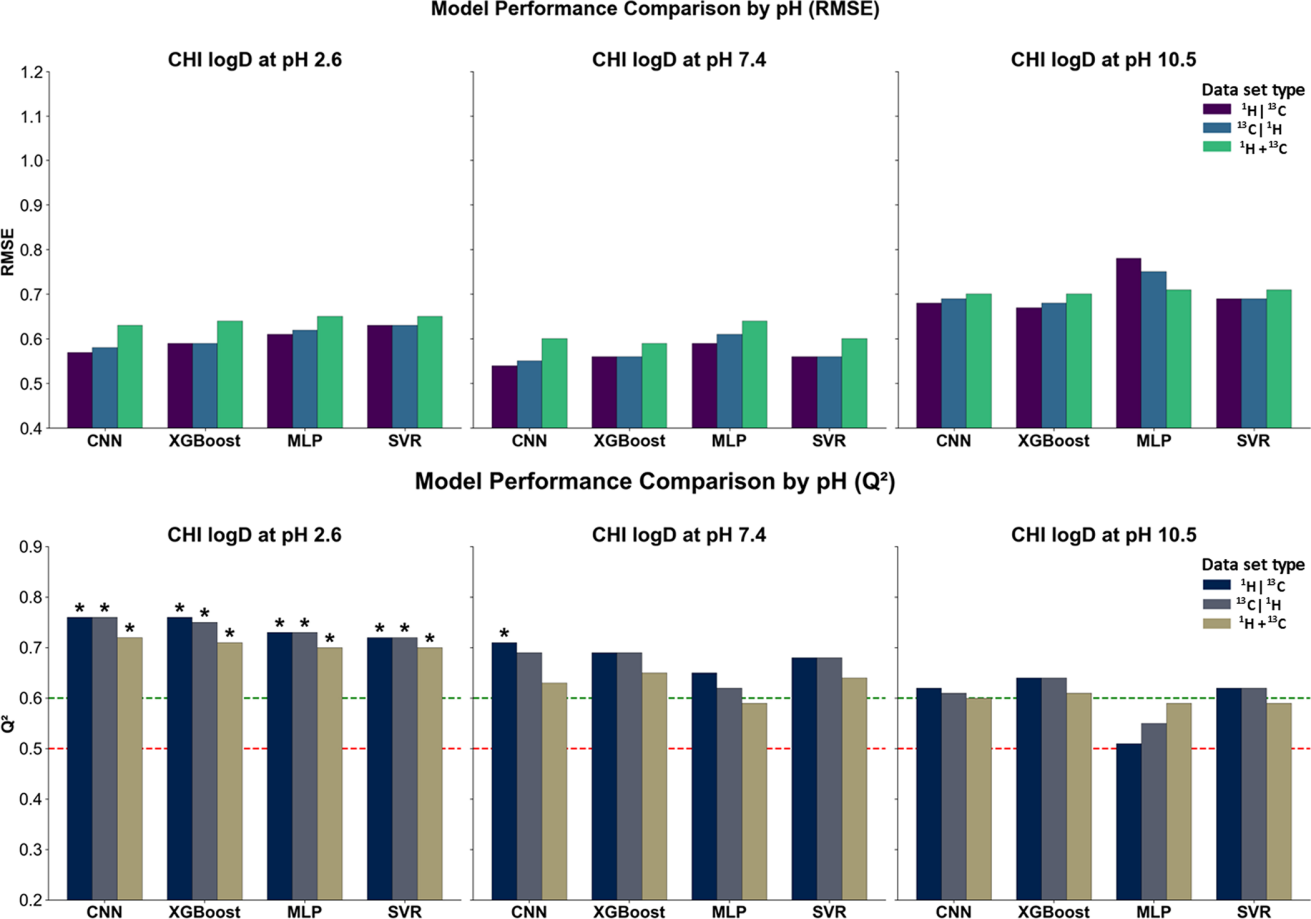
RMSE and *Q* ^2^ for models trained on fused spectral vectors created by concatenating or element-wise addition of ^1^H and ^13^C NMR representations. Each panel corresponds to a specific pH. Red and green lines mark *Q* ^2^ thresholds of 0.5 (minimal predictive value) and 0.6 (acceptable model), respectively; *Q* ^2^ ≥ 0.7 indicates a good model. Black asterisks above bars denote models exceeding this threshold.

At pH 7.4, CNN again led for ^1^H|^13^C vectors, reaching RMSE 0.54 and *Q* ^2^ 0.71, while both XGBoost and SVR followed closely with an RMSE of 0.56 and *Q* ^2^ values of 0.69 and 0.68. MLP remained the least performant, with *Q* ^2^ at 0.65. The same hierarchy persisted for the ^13^C | ^1^H setting, with negligible variation: CNN achieved a *Q* ^2^ of 0.69 (RMSE 0.55), and XGBoost and SVR reached *Q* ^2^ values of 0.69 and 0.68, respectively.

Under basic conditions (pH 10.5), all of the models showed a slight decline in predictive accuracy. XGBoost retained the lead for both ^1^H|^13^C and ^13^C|^1^H formats, with an RMSE of 0.67–0.68 and a *Q* ^2^ of 0.64. CNN and SVR followed with *Q* ^2^ values of 0.61–0.62 and RMSE values in the 0.68–0.69 range. MLP lagged consistently across all settings, scoring the lowest *Q* ^2^ of 0.51–0.55 at pH 10.5, regardless of fusion type.

An alternative approach, summing the ^1^H and ^13^C vectors (^1^H + ^13^C), yielded similar but slightly less favorable results. At pH 2.6, CNN and XGBoost achieved *Q* ^2^ values of 0.72 and 0.71, with RMSE values of 0.63 and 0.64, respectively. MLP and SVR followed in *Q* ^2^ at 0.70 with an RMSE of 0.65. At pH 7.4, performance remained tight across all models: CNN, XGBoost, and SVR achieved *Q* ^2^ between 0.63 and 0.65 with RMSE 0.59–0.60, while MLP trailed with a *Q* ^2^ of 0.59. At pH 10.5, the performance gap narrowed further, with all models reaching *Q* ^2^ around 0.59–0.61 and an RMSE of 0.70–0.71.

Importantly, the order of vector concatenation had minimal influence, as evidenced by near-identical metrics between ^1^H|^13^C and ^13^C|^1^H models (data not shown). All models trained on fused vectors showed strong convergence, with *R* ^2^_train consistently above 0.91 and frequently exceeding 0.96 (Figure S1). CNN and XGBoost maintained stable superiority across pH values, whereas MLP showed the weakest performance across all fusion schemes and conditions.

### Performance of Models Based on Hybrid Neural Networks

To further investigate the advantages of integrating both ^1^H and ^13^C NMR inputs, three hybrid neural network architectures were developed: CNN dual-stream and MLP dual-stream, both employing parallel processing of spectral branches, and a 2D CNN based on vertically stacked spectra. Performance was evaluated across three pH conditions (2.6, 7.4, and 10.5) (Figure 7).

**Figure 7:**
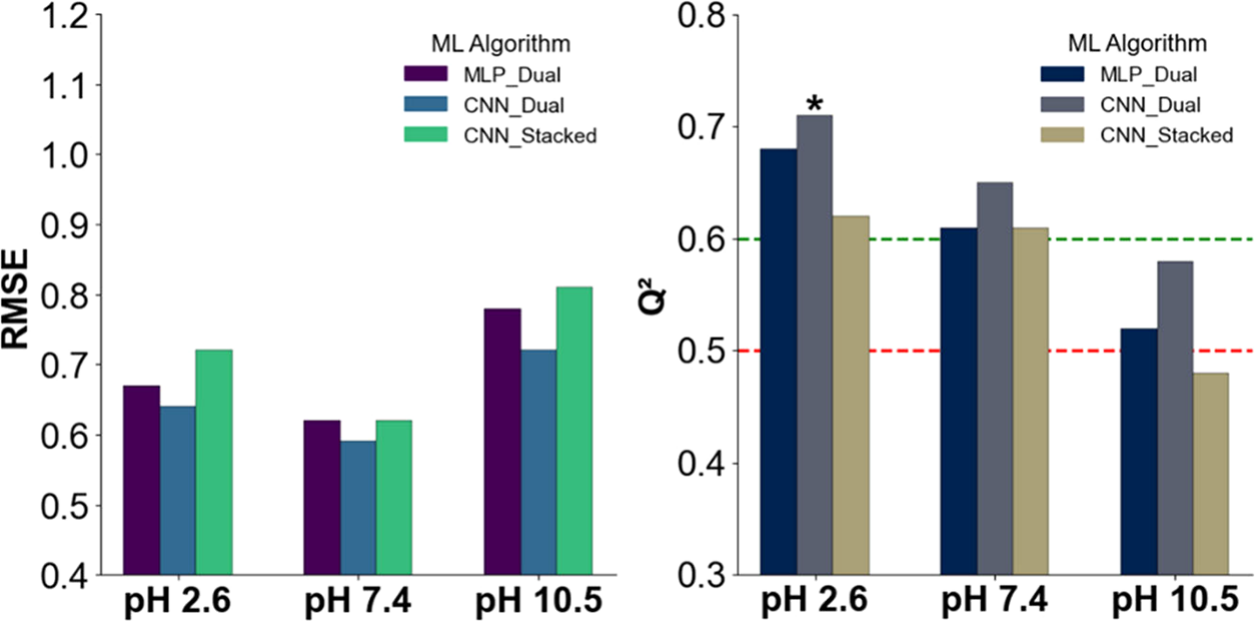
RMSE and *Q* ^2^ values for models trained on hybrid dual-stream (CNN and MLP) and 2D CNN stacked vector neural models at different pH levels. Red and green lines mark *Q* ^2^ thresholds of 0.5 (minimal predictive value) and 0.6 (acceptable model), respectively; *Q* ^2^ ≥ 0.7 indicates a good model. Black asterisks above bars denote models exceeding this threshold.

At pH 2.6, the CNN dual-stream model achieved the best prediction accuracy with an RMSE of 0.64 and a *Q* ^2^ of 0.71 (Figure 6), followed by MLP dual-stream (RMSE 0.67, *Q* ^2^ 0.68). The 2D CNN architecture performed slightly worse, reaching RMSE = 0.72 and *Q* ^2^ = 0.62. At pH 7.4, the same trend persisted: CNN dual-stream led with *Q* ^2^ 0.65 (RMSE 0.59), while both MLP and 2D CNN models followed closely, each reaching *Q* ^2^ values of 0.61 with an RMSE of 0.62. Under basic conditions (pH 10.5), predictive performance declined across all models: CNN dual-stream retained the lead with *Q* ^2^ 0.58 and RMSE 0.72, while MLP dual-stream reached *Q* ^2^ 0.52 (RMSE 0.78), and the 2D CNN trailed with *Q* ^2^ 0.48 and RMSE 0.81.

Despite relatively small numerical differences, the CNN dual-stream consistently outperformed the other architectures across all pH levels. Its design, which processes ^1^H and ^13^C branches independently using 1D convolutional filters before merging, appears particularly effective at capturing localized spectral features. MLP dual-stream, although lacking spatial awareness, benefited from the cross-attention mechanism and demonstrated robust generalization. In contrast, the 2D CNN using stacked spectra exhibited the weakest predictive power, possibly due to limited flexibility in capturing complex cross-dimensional patterns.

All hybrid models achieved *R* ^2^_train values exceeding 0.91, indicating a strong fit to training data (Figure S1). CNN and MLP dual-stream architectures were particularly stable, with *R* ^2^_train values up to 0.97 and 0.98, respectively, further confirming that dual-modality inputs can be effectively modeled using both spatial and attentional strategies.

### Prediction Based on Molecular Fingerprints (ECFP4)

To benchmark spectral representations against a cheminformatics standard, we trained models by using ECFP4. Each molecule was represented as a 2048-length binary vector derived from its SMILES code. Four ML architectures—SVR, XGBoost, CNN, and MLP—were trained with hyperparameter optimization and evaluated at three pH levels (Figure 8).

**Figure 8:**
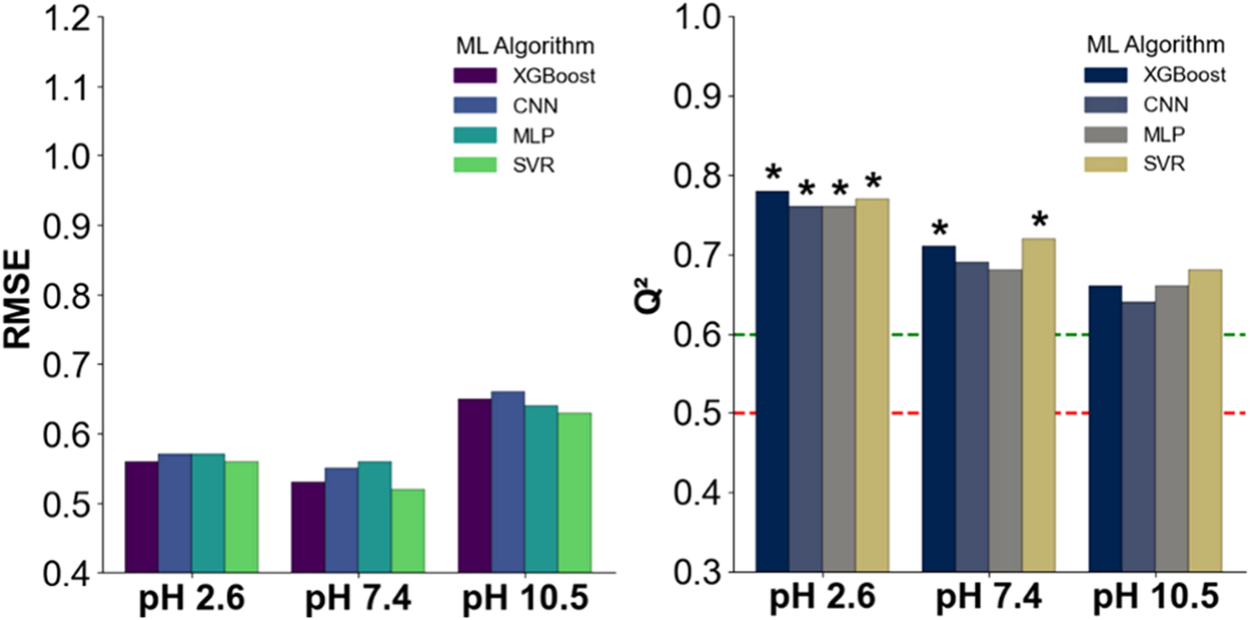
RMSE and *Q* ^2^ values for models trained on 2048-bit ECFP4 fingerprints derived from molecular SMILES codes. Red and green lines mark *Q* ^2^ thresholds of 0.5 (minimal predictive value) and 0.6 (acceptable model), respectively; *Q* ^2^ ≥ 0.7 indicates a good model. Black asterisks above bars denote models exceeding this threshold.

At pH 2.6, XGBoost and SVR yielded the best performance, both achieving RMSE 0.56 with *Q* ^2^ values of 0.78 and 0.77, respectively (Figure 6). CNN and MLP followed closely, each reaching RMSE of 0.57 and *Q* ^2^ of 0.76. At pH 7.4, SVR again led with RMSE 0.52 and *Q* ^2^ 0.72, while XGBoost and CNN produced similar results (RMSE 0.53–0.55, *Q* ^2^ 0.69–0.71). MLP performed slightly worse with RMSE 0.56 and *Q* ^2^ 0.68. At pH 10.5, performance dropped across the board: SVR remained slightly ahead with *Q* ^2^ 0.68 (RMSE 0.63), while MLP and XGBoost both scored *Q* ^2^ 0.66 with RMSE 0.64 and 0.65, respectively. CNN achieved *Q* ^2^ values of 0.64 and RMSE of 0.66.

Overall, ECFP4-based models maintained performance comparable to that of spectrum-driven models, with *Q* ^2^ values ranging from 0.64 to 0.78 across conditions. However, like all approaches tested, their predictive accuracy declined under basic conditions (pH 10.5). All optimized models exhibited an *R* ^2^_train above 0.90, often exceeding 0.95, confirming proper convergence during training (Figure S1). Among the evaluated algorithms, SVR and XGBoost showed the most consistent behavior, although no model architecture was entirely resilient to the drop in performance observed at high pH.

### Hyperparameter Importance Profiles

To evaluate optimization dynamics across different molecular representations, hyperparameter importance scores derived from Optuna optimization were analyzed.^26^ Further details are provided in the Supporting Information. This analysis covered all model types (SVR, XGBoost, MLP, CNN, and hybrid neural networks)—across four input representations: ^1^H NMR, ^13^C NMR, concatenated spectra (^1^H|^13^C), and ECFP4 fingerprints.

In SVR models, the γ parameter exhibited the highest importance when ^1^H and ^13^C spectra were used, often exceeding 0.70. For concatenated spectra and ECFP4, the importance of γ decreased, while epsilon increased above 0.25.

In XGBoost, γ (split loss reduction) and α (L1 regularization) were consistently dominant, with mean importance ≈0.42 and ≈0.40, respectively. Other hyperparameters (e.g., learning rate and max depth) had minimal influence (≤0.02).

In MLP models, learning rate was the most influential parameter (≈0.39), followed by the number of layers and dropout rate (each ≈0.13). Other parameters, such as batch size and number of units, showed low impact (≤0.09).

In CNN models, the learning rate remained the top contributor (up to 0.56), with dropout showing moderate influence. Kernel size, stride, and convolutional depth had low importance.

In hybrid neural networks, optimizer choice was the most impactful hyperparameter, particularly in the MLP dual at pH 2.6 (importance >0.80). Learning rate and dropout also played key roles. Cross-attention, when included, showed high importance at pH 10.5 (>0.70 in MLP dual). Other parameters had consistently low impact (<0.1).

### Applicability Domain

To evaluate the applicability domain (AD) of the trained models, Williams plots were generated for four representative cases. These were deliberately selected to reflect a wide range of architectural types, spectral input configurations, and pH conditions while also illustrating top-performing models within each category. The set includes (i) SVR trained on ^13^C spectra at pH 7.4—representing classical kernel methods applied to single-modality input; (ii) XGBoost on concatenated ^1^H|^13^C vectors at pH 2.6—illustrating ensemble methods on fused spectra; (iii) CNN trained on ^1^H data at pH 7.4—showcasing deep learning on proton-only input; and (iv) MLP dual-stream trained on ^1^H and ^13^C vectors at pH 10.5—representing complex dual-branch architectures under basic conditions. This diverse selection ensures that AD behavior is evaluated across input types, algorithmic families, and physicochemical contexts while grounding the analysis in models with strong predictive metrics. Each plot shows standardized residuals versus leverage for all training instances (Figure 9).

**Figure 9:**
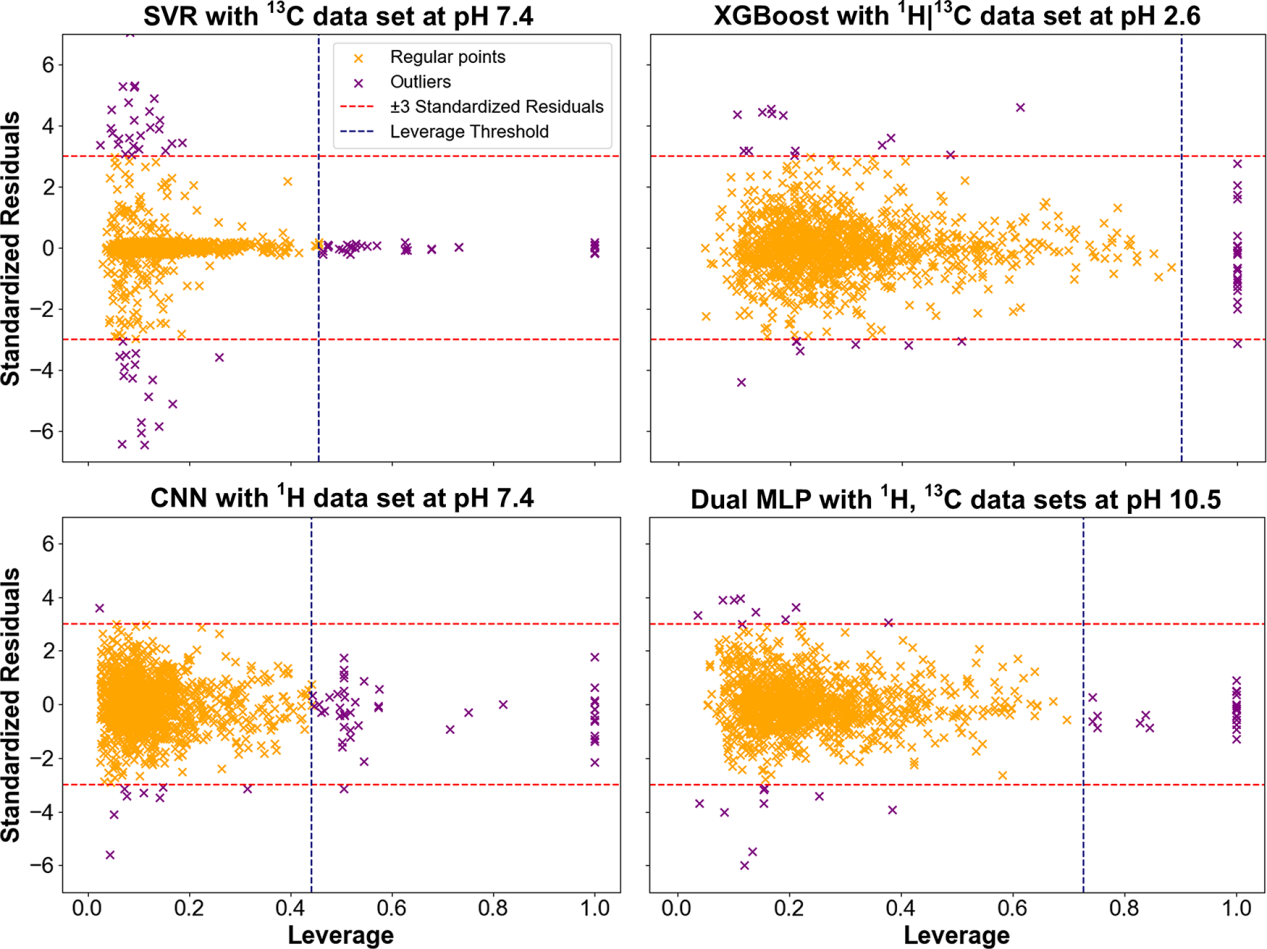
Williams plots for selected models illustrating the applicability domain of the CHI log *D* prediction. Four algorithms are shown: SVR (^13^C, pH 7.4), XGBoost (^1^H|^13^C, pH 2.6), CNN (^1^H, pH 7.4), and MLP dual-stream (^1^H + ^13^C, pH 10.5). Yellow dots: compounds within AD; purple: outliers (|residual| > 3 or leverage > h*). The *x*-axis corresponds to leverage values, and the *y*-axis to standardized residuals (common for all four subplots). The red dashed lines indicate ± 3 standardized residuals, and the blue dashed line marks the leverage threshold.

Across all models, most data points were located within the ±3 range of standardized residuals and below the calculated leverage threshold. The number of outliers ranged from 40 to 80, corresponding to 3.3–6.3% of the total training points. The SVR model had the highest outlier fraction (6.3%) and the widest residual span (−8.39 to 7.04). XGBoost showed the lowest outlier rate (3.3%) and a compact residual range (−4.38 to 4.61). CNN and MLP models yielded intermediate outlier fractions (4.4 and 3.3%) and moderately spread residuals. Leverage values for all models ranged from approximately 0.02 to 0.999.

## Discussion

### Comparative Overview of Spectral and Molecular Fingerprints

The comparative evaluation of input representations, ranging from classical ECFP4 fingerprints to ^1^H and ^13^C NMR spectra, their fused vectors, and dual-stream hybrids, provides a multidimensional perspective on the modeling of CHI log *D* across pH conditions. The best-performing models for each representation and pH level are summarized in Table 2, allowing a direct comparison of predictive quality across input types.

**Table 2:** Comparison of Best Model Performance (RMSE and *Q* ^2^) for Each Input Representation and pH^a^

CHI log *D* pH	metric	^1^H NMR (200)	^13^C NMR (200)	^1^H|^13^C Hybrid (2 × 200)^b^	^1^H|^13^C Fusion (400)^c^	ECFP4 (2048)
2.6	RMSE	0.64	0.62	0.64	0.57	0.56
	*Q* ^2^	0.71	0.72	0.71	0.76	0.78
7.4	RMSE	0.62	0.58	0.59	0.54	0.52
	*Q* ^2^	0.62	0.66	0.65	0.71	0.72
10.5	RMSE	0.74	0.69	0.72	0.67	0.66
	*Q* ^2^	0.57	0.60	0.58	0.64	0.64

a Values in parentheses indicate input vector length.

b ^1^H and ^13^C spectra, each encoded as a 200-dimensional vector, are supplied as two separate network inputs (2 × 200), i.e., not concatenated.

c ^1^H and ^13^C spectra, each encoded as a 200-dimensional vector, are concatenated into a single 400-dimensional input vector.

Across all pH conditions, the performance advantage of ECFP4 over fused spectral vectors was negligible, with *Q* ^2^ differences typically not exceeding 0.02. At pH 2.6, the best ECFP4 model (XGBoost) reached a *Q* ^2^ of 0.78 and an RMSE of 0.56, while the best fused vector model (CNN) achieved a *Q* ^2^ of 0.76 and an RMSE of 0.57. At pH 7.4, SVR trained on ECFP4 yielded a *Q* ^2^ 0.72 and an RMSE 0.52, compared to *Q* ^2^ 0.71 and RMSE 0.54 for CNN on fused spectra. At pH 10.5, both representations reached identical *Q* ^2^ values of 0.64, with RMSE values of 0.66 for ECFP4 and 0.67 for fusion. These marginal differences in predictive performance emphasize that NMR-based representations can reach levels of predictive accuracy similar to those of structural fingerprints. All models showed strong training performance, with *R* ^2^_train exceeding 0.94 across the board, confirming effective convergence and minimal underfitting. Williams plots indicated well-behaved residuals and a low fraction of outliers (typically <6.5%), supporting the robustness and generalization of all approaches. Thus, while ECFP4 remains a robust standard, it does not offer a decisive advantage in every scenario, especially given its much higher dimensionality and computational burden, which significantly amplifies training time in large-scale models.

Models based solely on ^1^H or ^13^C spectra underperformed their fused counterparts, but ^13^C vectors consistently outscored ^1^H in both RMSE and *Q* ^2^. This trend held at all pH levels and reflected the higher structural informativeness of ^13^C NMR for lipophilicity prediction. The superior performance of ^13^C-based models can be attributed to several intrinsic features of carbon NMR. First, the chemical shift range of ^13^C nuclei spans nearly 240 ppm, compared to just ∼12 ppm for ^1^H, offering a 20-fold increase in dispersion and thus facilitating the resolution of subtle structural differences. Second, carbon atoms exhibit greater structural diversity: while protons often occur in repetitive environments (e.g., −CH_3_, −CH_2_, and symmetrical aromatics), carbon skeletons contain numerous unique centers, including quaternary and aromatic carbons, which are especially abundant in druglike molecules. Moreover, ^13^C NMR captures deshielding effects mediated by extended π-systems and conjugation patterns, especially in polyaromatic and heterocyclic scaffolds, further enriching the representation space relevant for modeling properties such as lipophilicity.

Hybrid neural architectures that process ^1^H and ^13^C inputs in parallel exhibited mixed outcomes. While some achieved Q^2^ values close to or slightly above those of fused CNNs, they rarely improved the RMSE. For instance, at pH 2.6, the best hybrid model reached *Q* ^2^ 0.71 and RMSE 0.64, compared to *Q* ^2^ 0.76 and RMSE 0.57 for concatenated fusion (Table 2). This pattern held across other pH levels, suggesting that added architectural complexity does not necessarily enhance accuracy.

Among the evaluated algorithms, CNNs emerged as the most consistent top performer when applied to fused spectral representations. Across all pH levels, CNNs trained on concatenated ^1^H and ^13^C vectors achieved the best or near-best *Q* ^2^ and RMSE values, confirming their ability to effectively extract complementary features from dense, chemically meaningful inputs. This consistency positions CNNs as the preferred architecture for fused NMR-based modeling. However, this superiority does not fully extend to other spectral data types: for single-modality ^1^H or ^13^C inputs, as well as for hybrid dual-branch architectures, SVR and XGBoost often performed comparably or better (Table 2). Therefore, while CNNs offer an optimal balance of accuracy and scalability for fused spectral vectors, their dominance is architecture-specific and most pronounced when both the proton and carbon spectra are combined in a single representation.

In summary, fused NMR vectors provide an excellent trade-off between interpretability, predictive power, and computational efficiency. Although ECFP4 fingerprints remain a well-established benchmark in QSPR modeling and occasionally offer a slight edge in *Q* ^2^, the differences are minimal. Their high dimensionality—2048 sparse binary features—results in substantial computational overhead, particularly in neural networks. In contrast, fused spectral inputs are dense, compact (400 features), and yield nearly equivalent predictive performance across all pH conditions.

Interestingly, across all input types and modeling strategies, the highest predictive accuracy was consistently observed at pH 2.6, followed by that at pH 7.4, with the lowest performance at pH 10.5. This trend was evident regardless of representation, whether structural or spectral, and likely reflects intrinsic characteristics of the data set rather than limitations of specific models. Many druglike molecules are enriched in basic functional groups, such as aliphatic amines and nitrogen-containing heterocycles, which are predominantly protonated and well-characterized under acidic conditions. These functionalities are widely represented in medicinal chemistry due to their positive impact on aqueous solubility, permeability, and overall pharmacokinetic properties.^41,42^ In contrast, compounds bearing strongly acidic groups (e.g., carboxylic acids, sulfonates) are comparatively underrepresented, partly due to concerns regarding limited oral bioavailability and tissue penetration .^43,44^ Nonetheless, such groups are occasionally retained for their key roles in target-specific interactions, especially in binding sites requiring negative charge or hydrogen bonding.^43^ Overall, the observed bias toward basic functional groups reflects a balance between ADME-driven design principles and target-specific pharmacophores. As a result, molecular diversity at high pH may be lower or biased, with fewer molecules exhibiting reliable pH-dependent lipophilicity profiles. This compositional skew could reduce the richness of training data at pH 10.5 and partially explain the observed performance drop, independent of the input encoding strategy.

Sequential concatenation of ^1^H and ^13^C spectra consistently delivers strong results, making this approach a practical and scalable alternative to structural fingerprints in modern cheminformatics pipelines. Nevertheless, it is important to acknowledge that the predictive accuracy of models based on simulated NMR spectra strongly depends on spectral simulation fidelity. Factors such as overlapping signals, inaccuracies in chemical shift predictions, or representation of molecules containing nonstandard functionalities or heavy atoms may limit their reliability and generalizability.

### Spectral Interpretability and Physicochemical Attribution via SHAP Analysis

To gain insight into feature-level contributions in spectral models, a SHAP (SHapley Additive exPlanations)^45^ analysis of the best-performing convolutional neural network trained on concatenated ^1^H and ^13^C NMR spectra at pH 2.6 (Figure 10) was applied. Unlike abstract descriptors such as ECFP4, spectral features have direct physicochemical meaning, enabling transparent interpretation of model behavior in terms of real molecular environments. This direct traceability stands in contrast to conventional black-box models based solely on fingerprint encodings, which remain largely opaque and fall short of the transparency, reproducibility, and interpretability thresholds recently articulated by both the EMA and the FDA for AI-driven drug-development tools.^46,47^ To maintain clarity and focus, the analysis was restricted to the 30 most contributing features, ranked by the absolute SHAP value (Figure 10).

**Figure 10:**
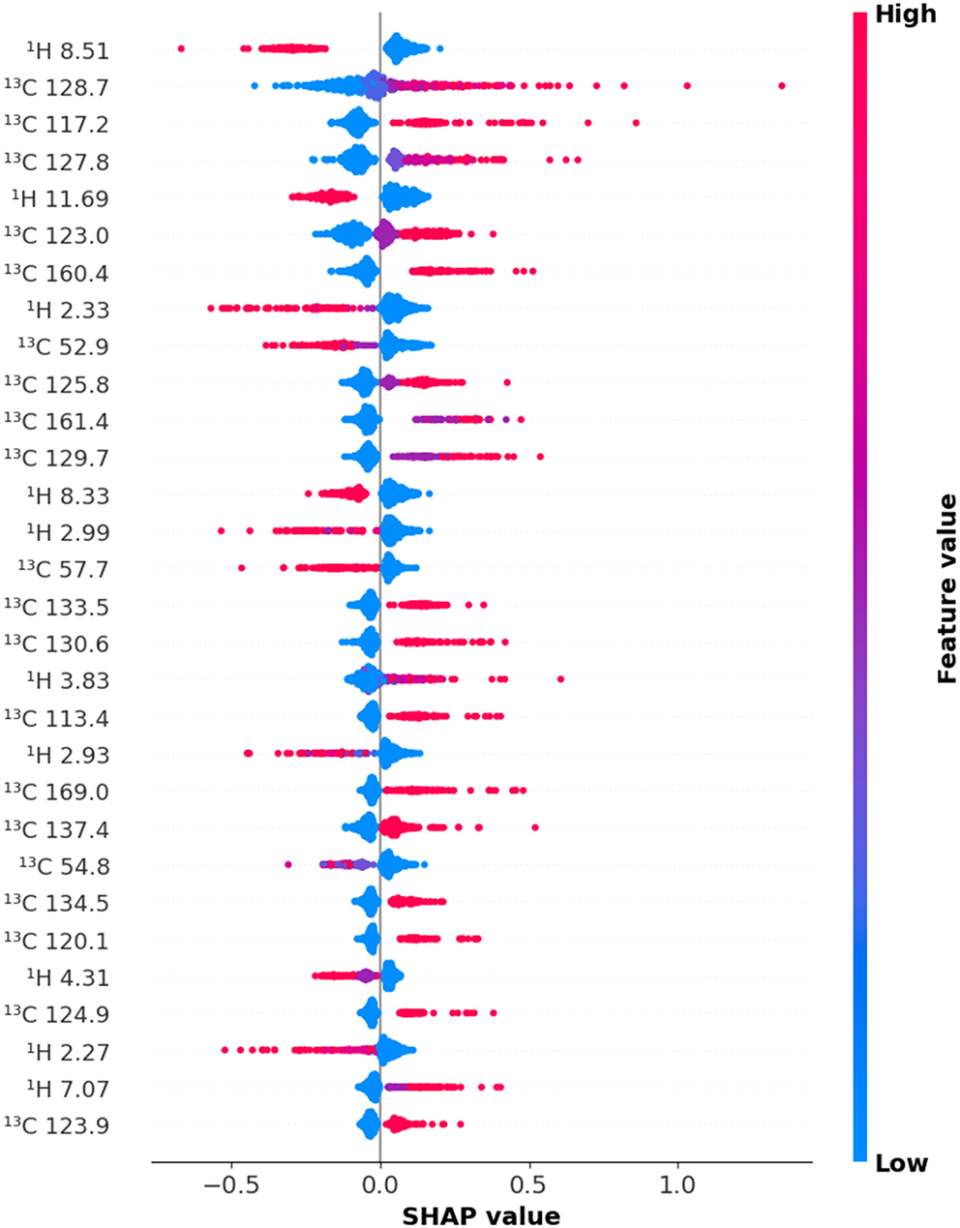
SHAP summary plot for the CNN model trained on fused ^1^H|^13^C NMR vectors at pH 2.6, displaying the 30 most contributing spectral features. Each row corresponds to *a* single bucket chemical shift centered at the indicated value. Bucket width is 0.06 ppm for ^1^H and 0.96 ppm for ^13^C. Points represent individual training instances; the *x*-axis indicates SHAP values (feature impact on predicted log *D*), and color denotes feature intensity (red = high, blue = low). Wider spreads reflect greater influence of the feature across the data set.

The SHAP plot reveals that increased signal intensity in the aromatic ^13^C region (110–150 ppm) correlates with higher predicted log *D*, consistent with the lipophilic character of aromatic systems. A similar positive contribution is observed for carbonyl-related signals between 150 and 170 ppm. Under acidic conditions (pH 2.6), partial protonation reduces the intrinsic polarity of these groups, increasing lipophilicity and thus explaining their positive SHAP contribution.

Negative contributions to log *D* prediction originate primarily from ^1^H features between 2.2 and 4.5 ppm, associated with hydroxyls, ethers, amines, and amides, moieties that increase polarity. These are reinforced by ^13^C signals in the 50–60 ppm region, typically arising from sp^3^ carbons bonded to electronegative atoms. Additional downshifts near 8 ppm in ^1^H NMR suggest contributions from heterocyclic NH or amide protons—also known to reduce lipophilicity.^48^

A key observation is the complementarity between modalities. ^1^H spectra capture dynamic, polar groups that are underrepresented in ^13^C NMR. The fused ^1^H|^13^C vector thus provides a more complete description of molecular environments, explaining its superior predictive performance. This chemical complementarity, not simply increased dimensionality, underpins the success of fused spectral models.

Unlike traditional fingerprints, spectral representations retain their physical links to the predicted property. SHAP analysis confirms that the CNN model captures structure–property relationships rooted in chemical reality, not just statistical optimization. Additional SHAP plots for pH 7.4 and 10.5 (Figures S2 and S3) show consistent patterns, supporting the robustness of this approach across varying protonation states.

### Spectral Representations Characterized by Hyperparameter Sensitivities

Hyperparameter optimization revealed distinct response patterns for models trained on NMR-based spectral inputs. Unlike ECFP4 fingerprints, sparse, binary, and structurally uniform, spectral vectors are dense, continuous, and semantically structured, leading to consistent dependencies across model classes.

In SVR, the γ parameter showed consistently high importance (>0.70) for both ^1^H and ^13^C spectra, reflecting strong sensitivity to locality and nonlinearity in spectral data. For fused and fingerprint inputs, γ’s influence decreased, while epsilon gained relevance, suggesting that tolerance margins play a greater role in broader or noisier feature spaces.

XGBoost models trained on spectral vectors highlighted γ (loss reduction) and α (L1 regularization) as dominant hyperparameters (≈0.42 and 0.40, respectively). These trends point to the need for pruning and sparsity control in concatenated inputs, where overlapping features may occur. Subsampling-related parameters contributed slightly, consistent with the deterministic structure of the spectral features.

In MLPs and CNNs, the learning rate consistently dominated (importance 0.39–0.56), followed by dropout and network depth. Surprisingly, convolution-specific parameters such as kernel size or number of filters had minimal impact, indicating that aligned and smooth spectra benefit more from postconvolutional processing than spatial abstraction.

Hybrid architectures—despite structural complexity—exhibited similar optimization patterns. Learning rate and optimizer type remained most influential, while structural elements such as cross-attention, kernel configuration, or embedding size had a negligible effect. An exception was observed at pH 10.5, where cross-attention in MLP dual gained importance (>0.70), suggesting potential relevance of intermodal coupling under specific chemical conditions.

Overall, hyperparameter profiles across models converged on a common principle: spectral vectors respond less to architectural complexity and more to training dynamics and regularization. Their continuous, dense structure not only favors smooth optimization landscapes but also demands fine-tuned control to mitigate overfitting. This distinguishes them from ECFP4 fingerprints, which, while computationally demanding, are more resilient to hyperparameter shifts.

These findings position NMR-based inputs as a distinctive representational regime, deterministic and information-rich, requiring careful calibration of learning behavior rather than exhaustive architectural exploration. Their robustness and efficiency offer a valuable alternative between rigid descriptors and learned embeddings.

### Log *D* Predictor

To facilitate practical applications of the developed models, all regression pipelines constructed in this work, namely, those based on ^1^H NMR, ^13^C NMR, fused (^1^H|^13^C), and molecular fingerprints, trained using SVR, XGBoost, CNN, and MLP architectures across three distinct pH values, were integrated into a single user-friendly prediction tool with a graphical interface. The resulting software is freely available via GitHub.

## Conclusions

This study demonstrates that NMR-derived spectral representations, when systematically processed and integrated into data-driven modeling pipelines, constitute a viable and robust alternative to conventional molecular fingerprints, offering comparable or slightly superior predictive performance across various log *D* modeling tasks. By transforming predicted ^1^H and ^13^C spectra into compact numerical vectors, we demonstrated that chemical shifts can encode molecular structure in a dense, information-rich format suitable for predictive modeling. Unlike conventional fingerprints, such as ECFP4, which rely on abstract graph topologies and atom environments, spectral descriptors offer a physically grounded alternative that reflects electronic distribution and chemical context directly.

A key insight of this work is that sequential fusion of ^1^H and ^13^C NMR spectra, implemented as straightforward vector concatenation, yields the most powerful predictive representations across all tested conditions. While ^13^C data alone consistently outperformed ^1^H and demonstrated clear underutilization in prior modeling efforts, it is the combined spectral encoding that delivered the highest overall accuracy. Notably, this low-dimensional, physically grounded fusion strategy outperformed more sophisticated dual-stream neural networks, suggesting that the architectural complexity does not guarantee better generalization. These findings challenge the commonly held assumption that deep neural integration of modalities is inherently superior and instead highlight the value of well-structured, interpretable representations grounded in chemical physics. This resonates with recent observations in cheminformatics suggesting that, especially in data-constrained regimes, simpler architectures can rival or even outperform their more complex counterparts, regardless of being neural or classical in nature.^49^

Notably, spectral vectors proved to be inherently low-dimensional yet semantically dense, enabling efficient model training without the computational overhead associated with fingerprints. At just 200–400 elements—up to an order of magnitude smaller than the 2048-bit ECFP4 fingerprints, these spectral vectors retain comparable predictive accuracy while slashing memory demands and training time. This has practical implications for scaling ML models across large compound libraries or in resource-constrained environments.

Beyond performance metrics, the analysis of hyperparameter importance and applicability domains revealed consistent behavioral patterns across model classes. Spectral inputs require fine control over kernel sharpness, regularization, and learning rate, underscoring their deterministic yet sensitive structure. These characteristics position them as ideal candidates for controlled modeling, where interpretability, data structure, and model behavior are tightly interlinked.

SHAP-based analysis confirmed that spectral inputs are not only predictive but also chemically meaningful, unveiling consistent associations between distinct spectral regions and molecular features, such as polarity and aromaticity. Consequently, NMR-driven models move beyond opaque prediction, offering regulator-grade traceability: spectral features map directly to molecular environments, supporting chemically intuitive derivation of reactivity, physicochemical traits, and ADME properties without the structural-fingerprint overhead of ECFP4. The historically weaker performance of models trained on ^1^H data alone highlights the importance of complementary modalities: proton-centered signals capture dynamic, polar functionalities but miss the carbon-based electronic signatures—aromatic and carbonyl resonances—readily resolved in ^13^C spectra. Together, these findings confirm that fused ^1^H|^13^C spectral vectors capture complementary chemical information, enabling accurate and interpretable logD prediction while satisfying emerging regulatory expectations for mechanistic transparency.

Finally, by integrating the best-performing models into a ready-to-use graphical tool, this work bridges the gap between algorithmic innovation and practical utility. For the first time, NMR spectra are not just tools of structural elucidation but actionable, predictive features in a cheminformatics pipeline.

This study positions NMR-based spectral modeling as a credible, scalable, and interpretable alternative to traditional cheminformatics approaches, offering new methodological options for both academic and industrial needs. The novelty lies not only in the successful use of predicted spectra but also in demonstrating that the informational content of NMR data, long confined to the domain of structural elucidation, can be systematically exploited for property prediction at scale. Given the generality and chemical interpretability of the approach, such spectral representations may be particularly valuable in early-stage drug discovery, not only for predicting a wide range of physicochemical and biochemical properties but also for informing scaffold selection, lead optimization, and ADME profiling.

## Supplementary Material



## Data Availability

All scripts, data files, and model configurations used throughout this study are publicly available under the MIT License. The complete repository containing all input data—including preprocessed spectral representations and molecular fingerprints—along with Python scripts for data preparation, model training, and evaluation is hosted at https://github.com/Prospero1988/NMR-AI_part3. In addition, the dedicated software *Demiurge*, used for automated spectral vector generation from SMILES strings, is available at https://github.com/Prospero1988/Demiurge. The log *D Predictor* application described in the final section— providing a user interface for model deployment and prediction—is accessible at https://github.com/Prospero1988/logD_predictor. The predictor is implemented in Python but features a graphical user interface (GUI) that requires no programming experience. Users can simply upload a. csv file containing SMILES strings, select the desired models and prediction options, and instantly obtain log *D* predictions. The application supports different verbosity levels, allowing users to adjust the amount of information returned, from minimal outputs to comprehensive prediction logs. Additionally, the tool generates summary plots visualizing the mean predicted log *D* values across selected models, along with standard deviations representing the consistency of the predictions.

## Notes

Complete tabular results (RMSE, *Q* ^2^, and *R* ^2^_train) for all machine learning models across multiple molecular representations and pH conditions (Tables S2–S6); data set summary and structural clustering overview; hyperparameter importance scores from Optuna (Tables S7–S11); *R* ^2^_train charts; optimization ranges for all model parameters (Table S12); detailed descriptions of neural network architectures; assessment of predictive model performance; Optuna hyperparameter’s importance description; and configuration details of the RBF kernel used in SVR models (PDF)

## Abbreviations

10CV: 10-fold cross-validation
^1^H NMR: proton nuclear magnetic resonance
^13^C NMR: carbon-13 nuclear magnetic resonance
AD: applicability domain
ADME: absorption, distribution, metabolism, and excretion
AI: artificial intelligence
CHI logD: chromatographic hydrophobicity index log *D*
CNN: convolutional neural network
ECFPs: extended-connectivity fingerprints
FC: fully connected
GUI: graphical user interface
HOSE: hierarchical organization of spherical environment codes
HPLC: high-performance liquid chromatography
log *D*: logarithm of the distribution constant
MACCS: molecular access system fingerprints
ML: machine learning
MLP: multilayer perceptron
NMR: nuclear magnetic resonance
NMRshiftDB2: nuclear magnetic resonance shift database
Optuna: hyperparameter optimization framework
pH: potential of hydrogen
PyTorch: python-based deep learning library
*R* ^2^: coefficient of determination
rbf: radial basis function
RMSE: root-mean-squared error
SD: standard deviation
SiLU: sigmoid linear unit
SMILES: simplified molecular input line entry system
SVR: support vector regression
XGBoost: extreme gradient boosting
