# Supplementary material for: From NMR to AI: Fusing 1H and 13C Representations for Enhanced QSPR Modeling
Source: J Chem Inf Model. 2025 Sep 17;65(19):10323–37. doi: 10.1021/acs.jcim.5c01791 (PMC12529761; doi:10.1021/acs.jcim.5c01791)
Supplement: Supplementary file 1 [file ci5c01791_si_001.pdf]

SUPPLEMENTARY INFORMATION

From NMR to AI: Fusing ^1H and ^{13}C Representations for Enhanced QSPR Modeling

Arkadiusz Leniak,[1] *Wojciech Pietruś,*[2*] *Rafał Kurczab*[2*]

[1] Department of Medicinal Chemistry, Celon Pharma S. A., ul. Marymoncka 15, 05-152 Kazun Nowy, Poland

[2] Department of Medicinal Chemistry, Maj Institute of Pharmacology, Polish Academy of Sciences, Smetna 12, 31-343 Krakow, Poland.

TABLE OF CONTENTS

DESCRIPTIONS: 2
Demiurge - Extended description 2
Details of the RBF kernel used in SVR models 4
Neural Networks - Extended description 5
Description of Hybrid Neural Networks 5
Hyperparameter importance scores 7
FIGURES: 9
Figure S1. R^2_train charts for all models across all representations and pH conditions. 9
Figure S2. SHAP chart for 1D CNN, $^1H|^{13}C$ dataset at pH 7.4 10
Figure S3. SHAP chart for 1D CNN, $^1H|^{13}C$ dataset at pH 10.5 11
TABLES: 12
Table S1. Dataset Summary and Structural Clustering Overview 12

Table S2. ^{1}H NMR models (RMSE, Q^2, and R^2_train)....................14
Table S3. ^{13}C NMR models (RMSE, Q^2, and R^2_train)....................15
Table S4. Fused ^{1}H/^{13}C NMR models (RMSE, Q^2, and R^2_train)....................16
Table S5. Hybrid neural ^{1}H/^{13}C NMR models (RMSE, Q^2, and R^2_train)....................17
Table S6. ECFP4 models (RMSE, Q^2, and R^2_train)....................18
Table S7. Hyperparameter importance values for SVR models....................18
Table S8. Hyperparameter importance values for XGB models....................20
Table S9. Hyperparameter importance values for MLP models....................21
Table S10. Hyperparameter importance values for CNN models....................22
Table S11a. Hyperparameter importance for MLP Dual-Stream models....................23
Table S11b. Hyperparameter importance for CNN Dual-Stream models....................23
Table S11c. Hyperparameter importance for 2D CNN models....................24
Table S12. Overview of all optimized Hyperparameters....................25
REFERENCES....................26

DESCRIPTIONS:

Demiurge - Extended description

To automate the generation of machine learning input data based on both simulated spectral and structural molecular representations, a software platform Demiurge, was implemented in Python. The full source code is publicly available on GitHub.[1] Demiurge processes input data in the form of .csv files containing compound structures encoded as SMILES strings along with the associated target values (in this work, CHI logD), and produces ready-to-use feature matrices tailored for both classical ML algorithms and neural networks. The software supports three types of molecular representations: predicted ^{1}H NMR spectra, predicted ^{13}C NMR

spectra, and ECFP4[2] molecular fingerprints. The initial module of Demiurge validates the input file, checks the syntax and presence of valid SMILES, and performs automatic correction of minor errors whenever feasible. Molecules are then reconstructed using the RDKit[3] library and saved as .mol files. Structures are initially optimized in three dimensions and then flattened to two dimensions by setting the z-coordinates to zero, ensuring compatibility with downstream NMR prediction tools. For the prediction of ^{1}H and ^{13}C NMR spectra, Demiurge employs a Java-based standalone predictor built upon the NMRshiftDB2 database.[4–6] This tool utilizes HOSE-code[7] pattern matching to assign averaged chemical shifts to atoms, referencing a precompiled .csv file containing the complete set of HOSE codes with their respective shift values. The predictor[8] internally calls the *org.openscience.nmrshiftdb* Java class and is executed locally via Python-to-Java system integration. The output consists of lists of predicted chemical shift values corresponding to individual H or C atoms in the molecule. Since these lists are not conventional NMR spectra, but instead an ordered set of shift values, a dedicated bucketing strategy was implemented to transform them into fixed-length numerical vectors suitable for machine learning models. A custom module was developed to divide the relevant chemical shift range into uniformly spaced bins. In this study, the ^{1}H NMR region (–1 to 16 ppm) and the ^{13}C NMR region (–10 to 230 ppm) were each divided into 200 equal-width buckets using a previously developed approach. Initially, each bucket is set to zero, and the predicted chemical shifts are iteratively assigned to their respective bins. The intensity value of a bin increases by one for each atom assigned to that bucket. For example, three equivalent protons from a methyl group would contribute a value of 3 to the corresponding bucket, while two protons from a methylene group would result in a value of 2. This process ensures that all molecular spectra are transformed into vectors of identical dimensionality, regardless of the number or type of

atoms present in the molecule. As an alternative structural representation, Demiurge also supports the generation of Extended Connectivity Fingerprints (ECFP4) using RDKit's Morgan fingerprinting algorithm with a radius of 2 (2048 bits). The resulting fingerprints are 2048-bit binary vectors, commonly used in cheminformatics and QSAR/QSPR modeling. After feature generation, the representations are merged with the corresponding CHI logD labels, headers are appended, and the resulting input matrices are saved as structured .csv files. All modules are optimized for parallel computation using 8 CPU cores, enabling efficient processing of large datasets. For a benchmark set of 1000 compounds, the average processing time is approximately 6 minutes for ^{1}H NMR spectra, 15 minutes for ^{13}C spectra, and under 2 minutes for RDKit fingerprints. The architecture of Demiurg is fully modular and extensible, allowing for straightforward adaptation to other endpoints such as logP, TPSA, or aqueous solubility (logS), as well as to alternative spectral formats.

Details of the RBF kernel used in SVR models

The RBF kernel was applied in SVR due to its ability to model nonlinear relationships by implicitly mapping spectral vectors into a high-dimensional feature space using the kernel trick, enabling the model to learn local dependencies without explicit transformation.[9] XGBoost was selected over classical Gradient Boosting due to its native GPU support and advanced algorithmic enhancements, including regularization mechanisms and second-order gradient optimization, which collectively yield superior model accuracy and training efficiency. These features proved essential given our high-throughput tuning approach relying on thousands of model evaluations. Indeed, comparative studies affirm that XGBoost offers both faster convergence and better generalization compared to conventional implementations.[10,11]

Neural Networks - Extended description

MLPs consisted of fully connected feedforward layers with nonlinear activations, optional dropout regularization, and batch normalization. These architectures are particularly well-suited for low-dimensional tabular inputs and served as the baseline deep learning models throughout this study.

In contrast, **CNNs** were employed to exploit the spatial continuity of spectral data. One-dimensional CNNs (1D CNNs) applied stacked 1D convolutional filters along the chemical shift axis to extract local peak features and neighborhood-level patterns. The extracted features were then aggregated through pooling and passed to fully connected layers to perform regression. In cases where spectral inputs were structured as two-channel matrices (e.g., combining ^{1}H and ^{13}C spectra), two-dimensional CNNs (2D CNNs) were used to allow convolutional filters to capture both intra- and inter-spectral correlations.

Description of Hybrid Neural Networks

The MLP Dual-Stream model consists of two independent feedforward branches, each dedicated to processing one of the NMR spectra (^{1}H or ^{13}C), represented as separate 200-element vectors. Each stream applies a customizable stack of fully connected (dense) layers with SiLU activation and dropout, transforming the raw inputs into latent embeddings of fixed dimension. To allow for flexible modeling of cross-nuclear interactions, the architecture optionally incorporates a cross-attention mechanism: the latent representation from the ^{1}H

stream is used as a query over the ^{13}C embedding (and vice versa), enabling each modality to selectively focus on informative features in the other. This bidirectional attention module captures potential correlations between proton and carbon environments that may be crucial for property prediction. The resulting attention-enhanced embeddings are then concatenated and passed through a shared fully connected regression head that outputs the final prediction. By combining the simplicity and expressiveness of MLPs with the relational modeling capabilities of attention, this architecture supports both modality-specific learning and cross-modal integration in a lightweight, interpretable framework.

The CNN Dual-Stream variant shares the same general dual-branch architecture but replaces fully connected layers with 1D convolutional blocks tailored for spectral data. Each NMR input ^{1}H or ^{13}C) is reshaped and passed through a dedicated sequence of convolutional layers, optionally including batch normalization, dropout, and non-linear activations. These convolutional pathways are designed to capture local patterns and positional dependencies inherent in NMR spectra. After global average pooling, the resulting embeddings can optionally be refined through the same cross-attention mechanism used in the MLP version, allowing each modality to selectively attend to features in the other. The final regression head processes the concatenated embeddings to predict the target property. This design emphasizes spatial sensitivity and feature locality, making it particularly suitable for recognizing consistent spectral motifs.

In contrast, the **CNN 2D Stacked-Spectra** architecture combined the ^{1}H and ^{13}C vectors vertically into a two-row matrix, yielding an input tensor of shape (1, 2, 200). Here, the convolutional filters first spanned both spectra vertically, allowing early interaction between ^{1}H and ^{13}C signals. Only the first convolutional layer used kernels of height 2, while all subsequent

layers operated along the chemical shift axis (height = 1), effectively reducing the tensor back to a single row representation. This structure enabled the network to learn localized joint motifs in the NMR input space.

In all three architectures, the number of layers, neurons, filter sizes, number of filters, dropout rates, and other architectural elements were not fixed manually. Instead, they were dynamically determined through full hyperparameter optimization using Optuna. Each model was trained independently at each pH level using the same input preparation pipeline and consistent 10-fold cross-validation protocols to ensure fair comparisons.

Hyperparameter importance scores

Hyperparameter importance scores derived from Optuna optimization provide a quantitative measure of how strongly individual hyperparameters influence the predictive performance of a machine learning model. These scores are calculated post hoc using functional analysis of variance (fANOVA) or similar methods implemented within the Optuna framework. During optimization, Optuna samples various hyperparameter configurations across the defined search space and records the associated objective function values, such as cross-validated RMSE. After the optimization is complete, the importance of each hyperparameter is assessed by estimating the marginal contribution of that parameter to the variation in model performance, while averaging out the effects of other parameters. This yields a relative score for each hyperparameter, normalized so that the total importance across all parameters sums to one.

The resulting importance profiles allow researchers to identify which aspects of the model's configuration have the greatest impact on performance, thus providing insight into model sensitivity and guiding future optimization efforts. For example, a high importance score for

the learning rate in neural networks indicates that small changes in this parameter have a large effect on predictive accuracy, whereas a near-zero score for dropout rate would suggest that regularization strength plays a minor role under the tested conditions. By analyzing these profiles, one can also uncover interactions between parameters or detect cases of overparameterization, where tuning certain variables yields minimal gain. In this study, hyperparameter importance scores were extracted for each algorithm–representation pair to better understand how different input types (e.g., spectral vs. structural) shape the model's response surface and optimization dynamics.

FIGURES:

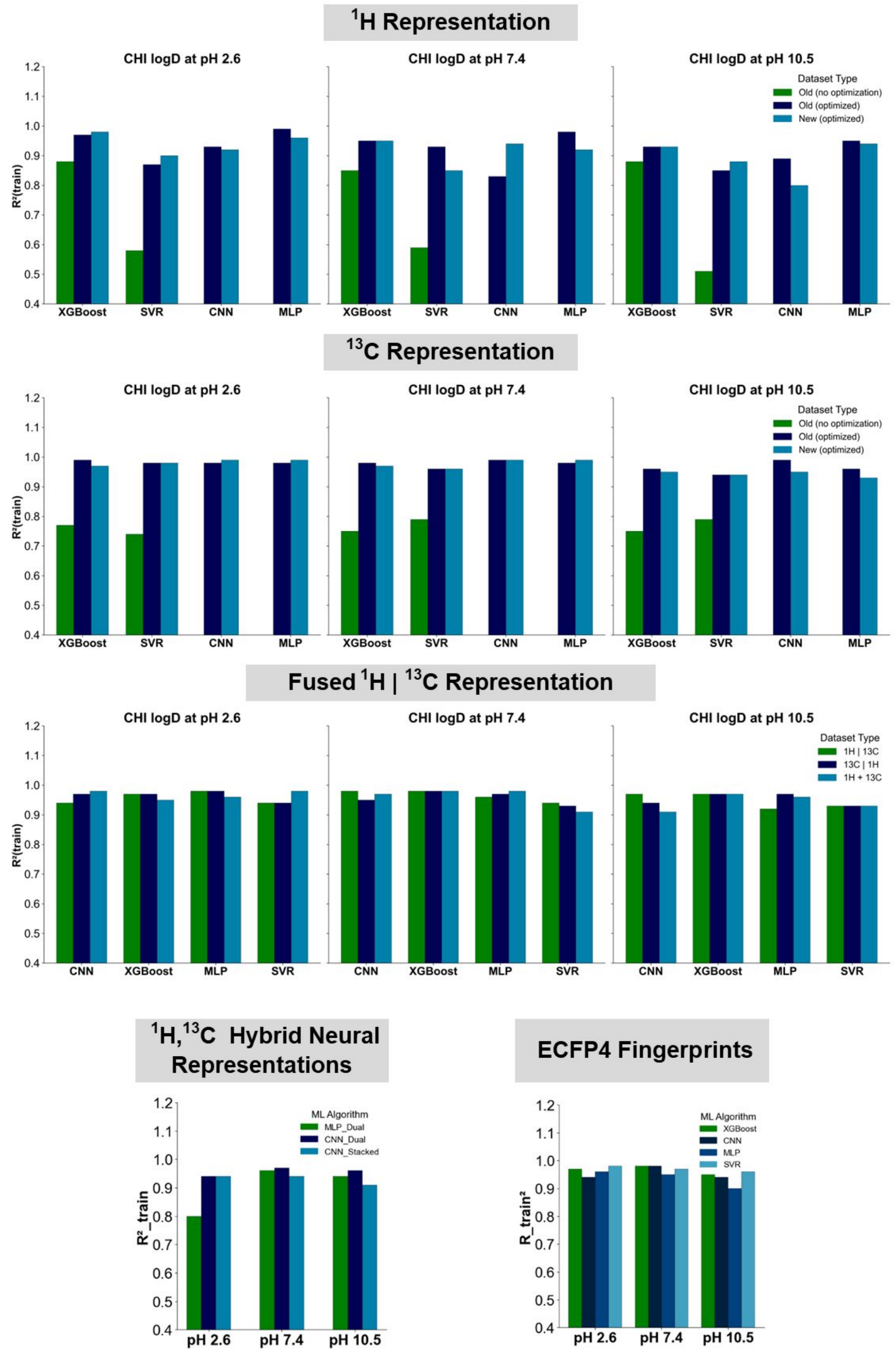

Figure S1. R^2_train charts for all models across all representations and pH conditions.

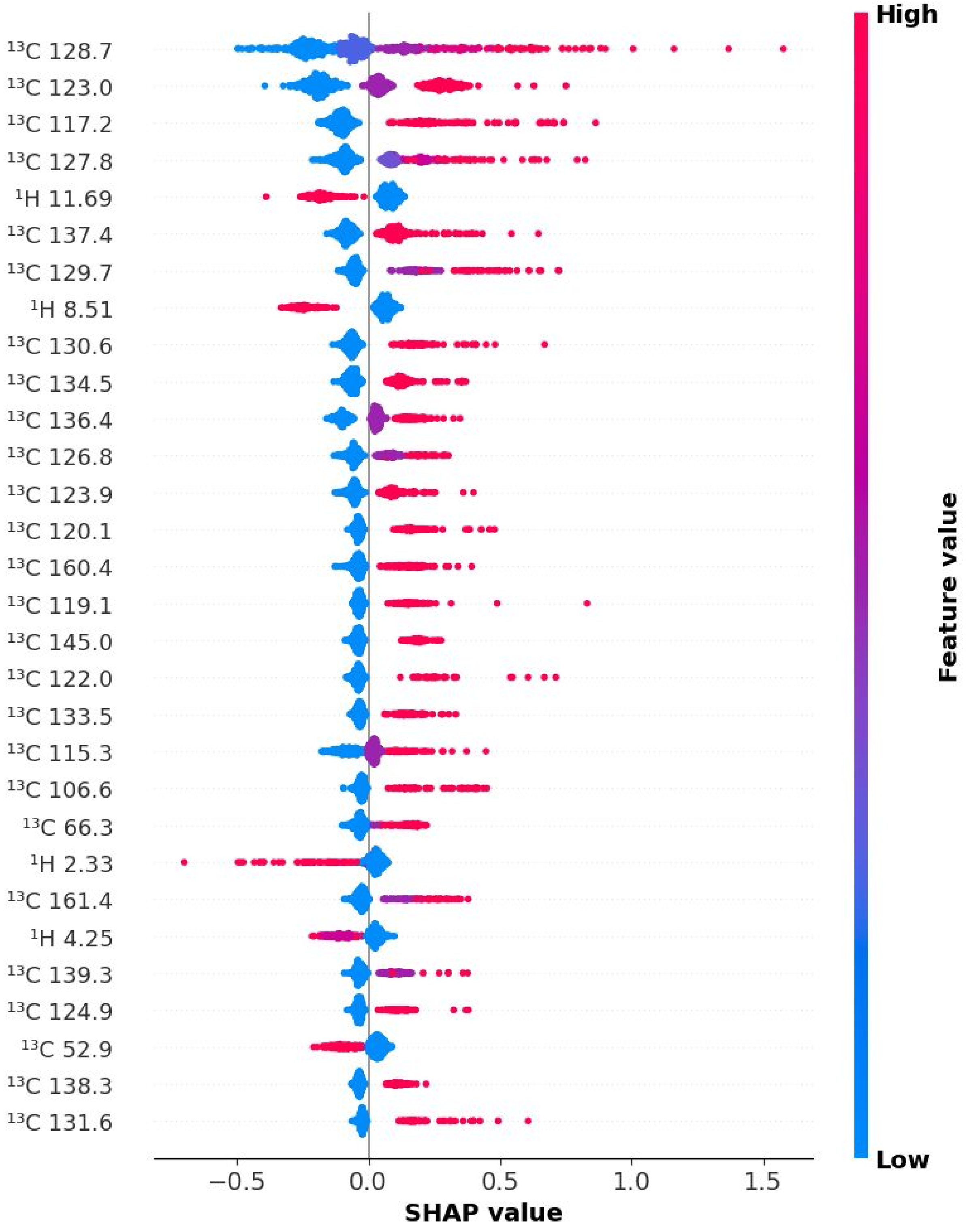

Figure S2. SHAP chart for 1D CNN, $^{1}H|^{13}C$ dataset at pH 7.4

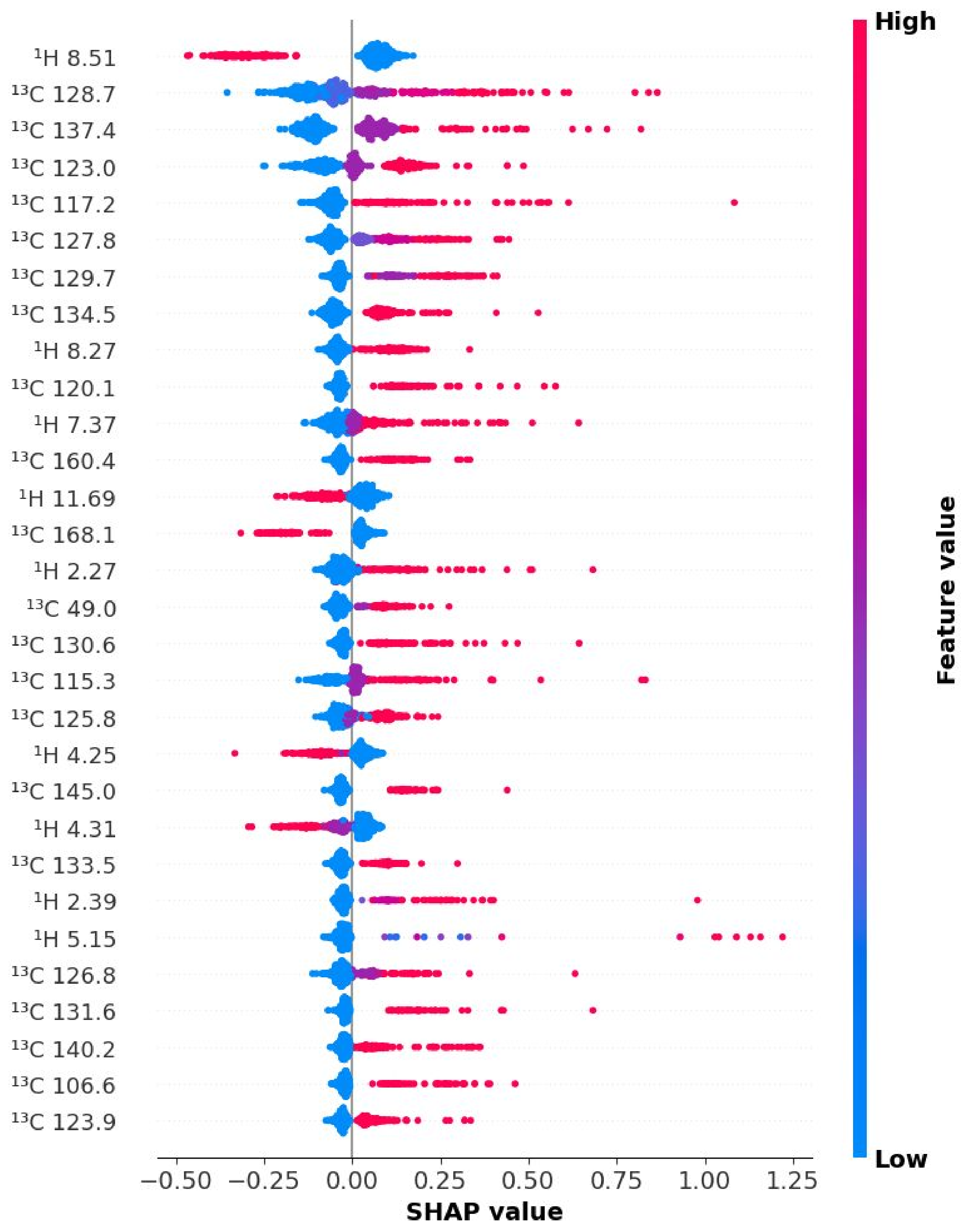

Figure S3. SHAP chart for 1D CNN, ^{1}H|^{13}C dataset at pH 10.5

TABLES:

Table S1. Dataset Summary and Structural Clustering Overview.

Summary of the consolidated dataset used for model development. The library contains 1290 aromatic compounds spanning a wide range of molecular weights and CHI-logD values across three pH conditions (2.6, 7.4, 10.5). Clustering based on ECFP4 fingerprints (Tanimoto cutoff = 0.75) yielded 43 chemotypes of variable size and diversity.

GROUP	MW_min	MW_max	logD2.6_min	logD2.6_max	logD7.4_min	logD7.4_max	logD10.5_min	logD10.5_max
1	1184.3	2790.4	2.49	4.29	2.76	4.59	1.25	4.80
2	234.2	307.4	0.92	3.31	0.74	3.24	2.66	3.22
3	184.2	464.5	-0.61	2.39	0.87	3.68	0.99	3.76
4	349.4	1021.3	0.24	3.46	2.44	3.57	2.39	3.94
5	347.4	603.5	-0.78	3.19	0.50	4.34	-0.04	5.73
6	307.7	620.1	-0.01	4.27	1.73	5.04	1.72	5.73
7	362.4	362.4	2.10	2.97	3.10	3.52	3.19	3.55
8	228.3	228.3	-0.25	-0.25	1.03	1.03	0.90	0.90
9	364.5	588.8	-0.58	1.39	0.51	4.03	1.12	4.53
10	464.6	477.4	0.54	0.95	2.02	3.12	2.11	2.53
11	397.5	593.7	0.49	1.17	2.27	3.80	4.03	4.62
12	320.4	568.6	-0.37	3.95	0.78	5.08	0.84	5.82
13	352.4	464.6	0.59	1.65	2.43	3.67	2.69	3.57
14	278.3	375.5	0.58	3.69	1.95	3.71	1.95	3.77
15	313.4	425.5	-0.55	1.56	0.72	3.80	0.88	3.93
16	382.3	382.3	-0.03	-0.03	0.63	0.63	1.09	1.09
17	290.3	616.6	-1.23	1.64	2.09	2.54	-0.57	2.62
18	450.3	450.3	0.19	0.19	1.88	1.88	2.96	2.96
19	441.5	441.5	1.88	1.88	2.35	2.35	2.35	2.35
20	437.5	527.5	1.32	3.77	1.94	3.72	0.65	2.61
21	396.5	396.5	0.05	0.05	1.52	1.52	1.19	1.19
22	338.5	352.5	0.28	0.73	1.16	1.95	1.23	1.93
23	400.5	605.8	3.07	3.80	3.01	4.89	3.10	5.64
24	238.3	481.7	1.24	1.58	3.42	4.47	3.23	4.17
25	382.5	382.5	0.80	0.80	2.56	2.56	3.54	3.54
26	214.2	392.9	2.47	3.86	2.44	3.80	2.48	3.85
27	279.3	369.4	0.66	3.56	1.64	3.52	0.53	3.58
28	369.5	369.5	0.14	0.40	1.94	2.06	2.16	2.25
29	248.3	503.8	-1.22	4.12	0.31	4.45	-0.45	5.82
30	288.4	380.5	-0.03	1.11	0.60	2.38	1.41	3.31
31	261.0	342.1	1.52	3.57	2.42	3.68	2.99	4.95
32	183.2	583.1	-1.36	3.36	0.22	4.02	-0.34	4.66
33	176.2	176.2					1.56	1.56
34	356.4	356.4	1.24	1.24	2.91	2.91	2.95	2.95

**35**	292.4	292.4	2.60	2.60	2.55	2.55	0.50	0.50
**36**	375.4	375.4	3.19	3.19	3.33	3.33		
**37**	278.4	459.4	-0.35	2.41	0.36	3.62	2.14	5.02
**38**	307.4	380.5	0.01	2.80	0.63	2.74	0.72	2.24
**39**	340.3	512.5	0.72	2.09	2.05	4.70	1.86	5.32
**40**	359.4	457.6	-0.32	0.54	1.00	2.30	1.50	4.36
**41**	351.5	470.5	-0.93	1.77	0.25	3.37	1.54	5.72
**42**	463.6	463.6	1.77	1.77	3.36	3.36	5.86	5.86
**43**	257.4	257.4	-0.10	-0.10	0.68	0.68	0.80	0.80

Table S2. ^{1}H NMR models (RMSE, Q^2, and R^2_train).

Full list of predictive performance metrics (RMSE, Q^2, and R^2_train) for each machine learning model trained on different dataset variants (Old – unoptimized, Old – optimized, and New – optimized), based on ^{1}H NMR spectral representations, at pH 2.6, 7.4, and 10.5. Each row corresponds to a unique model – dataset – pH combination.

**Dataset**	**pH**	**Model**	**RMSE**	**Q^2**	**R^2_train**
Old Dataset (no optimization)	2.6	**GradBoost**	**0.87**	0.54	0.88
		SVR	1.03	0.36	0.58
	7.4	**GradBoost**	**0.76**	0.43	0.85
		SVR	0.85	0.30	0.59
	10.5	**GradBoost**	**0.94**	0.43	0.88
		SVR	1.07	0.25	0.51
Old Dataset (optimized)	2.6	XGBoost	**0.78**	**0.62**	0.97
		SVR	0.84	0.55	0.87
		CNN	0.85	0.54	0.93
		MLP	0.90	0.48	0.99
	7.4	XGBoost	**0.72**	**0.58**	0.95
		CNN	0.76	0.52	0.83
		SVR	0.76	0.53	0.93
		MLP	0.80	0.47	0.98
	10.5	CNN	**0.85**	**0.57**	0.89
		XGBoost	0.86	0.56	0.93
		MLP	0.92	0.50	0.95
		SVR	0.92	0.50	0.85
New Dataset (optimized)	2.6	XGBoost	**0.64**	**0.71**	0.98
		CNN	0.66	0.68	0.92
		MLP	0.69	0.65	0.96
		SVR	0.69	0.66	0.90
	7.4	XGBoost	**0.62**	**0.62**	0.95
		CNN	0.64	0.58	0.94
		SVR	0.65	0.54	0.85
		MLP	0.66	0.56	0.92
	10.5	XGBoost	**0.74**	**0.57**	0.93
		CNN	0.76	0.53	0.80
		SVR	0.77	0.52	0.88
		MLP	0.81	0.47	0.94

Table S3. ^{13}C NMR models (RMSE, Q^2, and R^2_train).

Full list of RMSE, Q^2, and R^2_train values for models trained on ^{13}C NMR spectral data, comparing unoptimized and optimized training on both the original and expanded datasets across three pH levels. Each row corresponds to a unique model – dataset – pH combination.

Dataset	pH	Model	RMSE	Q^2	R^2_train
Old (no optimization)	2.6	**GradBoost**	**0.87**	0.52	0.77
		SVR	0.89	0.50	0.74
	7.4	**SVR**	**0.72**	0.58	0.79
		GradBoost	0.81	0.46	0.75
	10.5	**SVR**	**0.88**	0.55	0.79
		GradBoost	0.94	0.48	0.75
Old (optimized)	2.6	**CNN**	**0.72**	0.66	0.98
		XGB	0.76	0.63	0.99
		SVR	0.76	0.63	0.98
		MLP	0.78	0.60	0.98
	7.4	**CNN**	**0.63**	0.67	0.99
		SVR	0.65	0.65	0.96
		MLP	0.72	0.57	0.98
		XGB	0.68	0.61	0.98
	10.5	**CNN**	**0.77**	0.65	0.99
		SVR	0.78	0.63	0.94
		XGB	0.80	0.61	0.96
		MLP	0.90	0.51	0.96
New (optimized	2.6	**CNN**	**0.62**	0.72	0.99
		XGB	0.63	0.75	0.97
		MLP	0.64	0.71	0.99
		SVR	0.70	0.68	0.98
	7.4	**CNN**	**0.58**	0.66	0.99
		XGB	0.58	0.66	0.97
		MLP	0.59	0.65	0.99
		SVR	0.61	0.59	0.96
	10.5	**SVR**	**0.69**	0.60	0.94
		MLP	0.72	0.58	0.95
		CNN	0.72	0.58	0.95
		XGB	0.72	0.57	0.93

Table S4. Fused $^{1}H/^{13}C$ NMR models (RMSE, Q^2, and R^2_train).

Summary of predictive performance RMSE, Q^2, and R^2_train for all models trained using fused $^{1}H/^{13}C$ spectral vectors, evaluated across multiple pH levels. Both concatenated and additive fusion strategies were considered.

Vector type	pH	ML algorithm	RMSE	Q^2	R^2_train
1H | 13C	2.6	CNN	0.57	0.76	0.94
		XGB	0.59	0.76	0.97
		MLP	0.61	0.73	0.98
		SVR	0.63	0.72	0.94
	7.4	CNN	0.54	0.71	0.98
		XGB	0.56	0.69	0.98
		SVR	0.56	0.68	0.94
		MLP	0.59	0.65	0.96
	10.5	XGB	0.67	0.64	0.97
		CNN	0.68	0.62	0.97
		SVR	0.69	0.62	0.93
		MLP	0.78	0.51	0.92
13C | 1H	2.6	CNN	0.58	0.76	0.97
		XGB	0.59	0.75	0.97
		MLP	0.62	0.73	0.98
		SVR	0.63	0.72	0.94
	7.4	CNN	0.55	0.69	0.95
		XGB	0.56	0.69	0.98
		SVR	0.56	0.68	0.93
		MLP	0.61	0.62	0.97
	10.5	XGB	0.68	0.64	0.97
		CNN	0.69	0.61	0.94
		SVR	0.69	0.62	0.93
		MLP	0.75	0.55	0.97
1H + 13C	2.6	CNN	0.63	0.72	0.98
		XGB	0.64	0.71	0.95
		MLP	0.65	0.70	0.96
		SVR	0.65	0.70	0.98
	7.4	XGB	0.59	0.65	0.98
		SVR	0.60	0.64	0.91
		CNN	0.60	0.63	0.97
		MLP	0.64	0.59	0.98
	10.5	XGB	0.70	0.61	0.97
		CNN	0.70	0.60	0.91
		SVR	0.71	0.59	0.93
		MLP	0.71	0.59	0.96

Table S5. Hybrid neural $^1H/^{13}C$ NMR models (RMSE, Q^2, and R^2_train).

Summary of predictive performance (RMSE, Q^2, and R^2_train for all models trained using hybrid $^1H/^{13}C$ spectral vectors, evaluated across multiple pH levels. Results from neural networks with different tensors architecture.

ML algorithm	pH	RMSE	Q^2	R^2_train
CNN Dual-Stream	2.6	**0.64**	0.71	0.94
MLP Dual-Stream	2.6	0.67	0.68	0.80
2D CNN Stacked Vectors	2.6	0.72	0.62	0.94
CNN Dual-Stream	7.4	**0.59**	0.65	0.97
2D CNN Stacked Vectors	7.4	0.62	0.61	0.96
MLP Dual-Stream	7.4	0.62	0.61	0.94
CNN Dual-Stream	10.5	**0.72**	0.58	0.96
MLP Dual-Stream	10.5	0.78	0.52	0.94
2D CNN Stacked Vectors	10.5	0.81	0.48	0.91

Table S6. ECFP4 models (RMSE, Q^2, and R^2_train).

Summary of predictive performance (RMSE, Q^2, and R^2_train) for models trained on ECFP4 fingerprints, evaluated at three pH levels using four machine learning algorithms.

Vector type	CHI logD pH	ML algorithm	RMSE	Q^2	R^2_train
ECFP4	2.6	XGB	0.56	0.78	0.97
		CNN	0.57	0.76	0.94
		MLP	0.57	0.76	0.96
		SVR	0.56	0.77	0.98
	7.4	SVR	0.52	0.72	0.97
		XGB	0.53	0.71	0.98
		CNN	0.55	0.69	0.98
		MLP	0.56	0.68	0.95
	10.5	SVR	0.63	0.68	0.96
		MLP	0.64	0.66	0.90
		XGB	0.65	0.66	0.95
		CNN	0.66	0.64	0.94

Table S7. Hyperparameter importance values for SVR models.

Computed using Optuna's *get_param_importances()* method. Each row corresponds to a specific input representation and pH value. Importance scores reflect the relative contribution of each hyperparameter to model performance during optimization trials.

Dataset	CHI logD pH	gamma	C	tol	epsilon
13C	2.6	0.88	0.08	0.03	0.01
13C	7.4	0.43	0.41	0.14	0.02
13C	10.5	0.79	0.09	0.09	0.02
13C1H	2.6	0.29	0.30	0.13	0.27
13C1H	7.4	0.24	0.27	0.19	0.31
13C1H	10.5	0.75	0.12	0.12	0.01
1H	2.6	0.6	0.20	0.07	0.14
1H	7.4	0.68	0.11	0.19	0.01
1H	10.5	0.81	0.07	0.04	0.08
1H13C	2.6	0.53	0.16	0.23	0.09
1H13C	7.4	0.16	0.27	0.34	0.22
1H13C	10.5	0.43	0.44	0.06	0.08
1H+13C	2.6	0.69	0.11	0.08	0.12
1H+13C	7.4	0.72	0.09	0.18	0.01
1H+13C	10.5	0.77	0.09	0.13	0.01
FP	2.6	0.05	0.15	0.02	0.79
FP	7.4	0.16	0.11	0.37	0.37
FP	10.5	0.11	0.36	0.03	0.50

Table S8. Hyperparameter importance values for XGB models.

Complete importance profiles for all XGBoost models trained on various input types across pH 2.6, 7.4, and 10.5. Values were generated via Optuna's trial-based importance estimation and are presented per input – pH combination.

Dataset	pH	gamma	reg alpha	reg lambda	delta step	child weigth	colsampl e bynode	grow policy	subsamp le	learning rate	colsampl e bytree	colsampl e bylevel	max depth
13C	2.6	0.42	0.25	0.09	0.05	0.05	0.04	0.02	0.02	0.01	0.01	0.00	0.00
13C	7.4	0.70	0.19	0.00	0.00	0.00	0.03	0.00	0.00	0.00	0.02	0.02	0.00
13C	10.5	0.68	0.08	0.04	0.02	0.05	0.03	0.00	0.04	0.01	0.01	0.03	0.00
13C1H	2.6	0.36	0.55	0.00	0.01	0.01	0.01	0.00	0.02	0.00	0.00	0.00	0.00
13C1H	7.4	0.28	0.52	0.03	0.02	0.00	0.02	0.00	0.02	0.00	0.05	0.02	0.02
13C1H	10.5	0.41	0.48	0.00	0.02	0.02	0.01	0.00	0.00	0.01	0.00	0.02	0.01
1H	2.6	0.36	0.39	0.01	0.04	0.01	0.02	0.00	0.01	0.02	0.01	0.05	0.08
1H	7.4	0.61	0.25	0.00	0.04	0.00	0.02	0.00	0.00	0.02	0.02	0.00	0.00
1H	10.5	0.33	0.45	0.00	0.02	0.05	0.03	0.02	0.04	0.01	0.00	0.03	0.02
1H13C	2.6	0.39	0.50	0.00	0.01	0.02	0.01	0.00	0.00	0.01	0.00	0.02	0.03
1H13C	7.4	0.48	0.31	0.00	0.02	0.03	0.00	0.00	0.03	0.03	0.02	0.03	0.02
1H13C	10.5	0.43	0.34	0.01	0.06	0.02	0.03	0.01	0.02	0.02	0.03	0.04	0.00
1H+13C	2.6	0.49	0.18	0.05	0.06	0.03	0.05	0.00	0.07	0.04	0.00	0.02	0.02
1H+13C	7.4	0.4	0.49	0.00	0.00	0.01	0.01	0.01	0.00	0.00	0.03	0.00	0.01
1H+13C	10.5	0.43	0.48	0.00	0.00	0.00	0.03	0.00	0.00	0.03	0.00	0.00	0.01
FP	2.6	0.25	0.64	0.00	0.01	0.00	0.02	0.00	0.00	0.01	0.01	0.02	0.01
FP	7.4	0.20	0.51	0.00	0.00	0.00	0.00	0.00	0.00	0.00	0.00	0.04	0.00
FP	10.5	0.34	0.57	0.00	0.03	0.10	0.00	0.00	0.02	0.04	0.03	0.04	0.00

Table S9. Hyperparameter importance values for MLP models.

Detailed Optuna-derived hyperparameter importances for MLP architectures. Each row in the table corresponds to a specific spectral or fingerprint representation used at a given pH condition.

dataset	pH	learning rate	weight init	clip grad value	num layers	dropout rate	use batch norm	regularization	batch size	units	optimizer	early stop patience	activation	use scheduler	epochs
1H+13C	2.6	0.66	0.13	0.08	0.06	0.02	0.02	0.01	0.01	0.01	0.01	0.00	0.00	0.00	0.00
1H+13C	7.4	0.25	0.00	0.00	0.37	0.03	0.06	0.00	0.01	0.01	0.01	0.18	0.03	0.05	0.00
1H+13C	10.5	0.23	0.01	0.00	0.12	0.00	0.19	0.00	0.03	0.22	0.00	0.00	0.18	0.00	0.01
1H13C	2.6	0.00	0.00	0.00	0.19	0.05	0.00	0.00	0.58	0.17	0.00	0.00	0.00	0.00	0.00
1H13C	7.4	0.09	0.02	0.02	0.03	0.47	0.00	0.00	0.11	0.14	0.00	0.00	0.11	0.00	0.00
1H13C	10.5	0.91	0.00	0.00	0.00	0.01	0.00	0.01	0.00	0.01	0.00	0.04	0.01	0.00	0.00
13C1H	2.6	0.90	0.00	0.01	0.03	0.00	0.01	0.00	0.02	0.00	0.00	0.00	0.00	0.00	0.01
13C1H	7.4	0.45	0.00	0.00	0.20	0.00	0.00	0.00	0.00	0.27	0.04	0.03	0.00	0.00	0.00
13C1H	10.5	0.36	0.01	0.01	0.34	0.00	0.00	0.00	0.01	0.09	0.01	0.01	0.17	0.00	0.00
FP	2.6	0.05	0.00	0.16	0.01	0.03	0.08	0.00	0.59	0.01	0.03	0.03	0.01	0.00	0.01
FP	7.4	0.11	0.00	0.00	0.01	0.65	0.03	0.01	0.14	0.00	0.00	0.03	0.01	0.00	0.00
FP	10.5	0.03	0.00	0.02	0.01	0.02	0.10	0.00	0.10	0.42	0.25	0.01	0.01	0.02	0.00
1H	2.6	0.02	0.00	0.00	0.17	0.51	0.01	0.10	0.02	0.00	0.05	0.00	0.11	0.00	0.00
13C	2.6	0.66	0.00	0.00	0.08	0.20	0.00	0.00	0.02	0.00	0.01	0.01	0.00	0.01	0.00
1H	7.4	0.57	0.01	0.01	0.01	0.01	0.35	0.01	0.01	0.01	0.00	0.00	0.01	0.01	0.01
13C	7.4	0.91	0.00	0.00	0.01	0.02	0.00	0.02	0.02	0.01	0.00	0.00	0.00	0.00	0.00
1H	10.5	0.71	0.00	0.00	0.00	0.21	0.01	0.00	0.00	0.00	0.00	0.02	0.00	0.05	0.00
13C	10.5	0.16	0.00	0.02	0.65	0.01	0.00	0.03	0.01	0.01	0.05	0.01	0.01	0.02	0.02

Table S10. Hyperparameter importance values for CNN models.

Full listing of hyperparameter importance scores for CNN models optimized across different molecular representations and pH levels. Values reflect importance estimates calculated per optimization run using the Optuna framework.

dataset	pH	activation	batch size	clip grad value	dropout rate	early stop patience	epochs	fc units l0	fc units l1	kernel size l0	learning rate
13C	2.6	0.01	0.28	0.00	0.02	0.00	0.08	0.02	0.18	0.00	0.20
13C	7.4	0.00	0.00	0.00	0.05	0.03	0.00	0.07	0.01	0.00	0.79
13C	10.5	0.00	0.28	0.00	0.01	0.00	0.01	0.00	0.05	0.00	0.52
1H	2.6	0.13	0.01	0.02	0.01	0.03	0.03	0.00	0.28	0.02	0.24
1H	7.4	0.14	0.00	0.00	0.07	0.01	0.00	0.07	0.05	0.00	0.22
1H	10.5	0.05	0.00	0.00	0.12	0.01	0.00	0.24	0.00	0.01	0.04
1H13C	2.6	0.01	0.01	0.02	0.01	0.02	0.00	0.03	0.00	0.00	0.78
1H13C	7.4	0.00	0.00	0.02	0.00	0.00	0.00	0.01	0.03	0.01	0.70
1H13C	10.5	0.01	0.00	0.01	0.00	0.00	0.73	0.01	0.08	0.00	0.13
1H+13C	2.6	0.12	0.06	0.03	0.42	0.01	0.01	0.03	0.05	0.00	0.02
1H+13C	7.4	0.00	0.01	0.00	0.00	0.01	0.01	0.05	0.68	0.01	0.16
1H+13C	10.5	0.00	0.01	0.00	0.12	0.00	0.00	0.56	0.29	0.00	0.01
13C1H	2.6	0.01	0.01	0.00	0.00	0.04	0.00	0.03	0.05	0.03	0.56
13C1H	7.4	0.12	0.00	0.06	0.08	0.01	0.04	0.01	0.01	0.02	0.40
13C1H	10.5	0.02	0.01	0.05	0.00	0.05	0.01	0.31	0.00	0.01	0.05
FP	2.6	0.00	0.02	0.00	0.01	0.00	0.00	0.01	0.01	0.00	0.91
FP	7.4	0.00	0.00	0.00	0.00	0.01	0.05	0.04	0.06	0.00	0.71
FP	10.5	0.06	0.01	0.08	0.01	0.01	0.01	0.04	0.02	0.04	0.63

dataset	pH	num conv layers	num fc layers	num filters l0	optimizer	padding l0	regularization	stride l0	use batch norm	use scheduler	weight init
13C	2.6	0.02	0.00	0.00	0.13	0.00	0.02	0.00	0.01	0.01	0.03
13C	7.4	0.00	0.00	0.01	0.01	0.00	0.01	0.00	0.00	0.01	0.00
13C	10.5	0.00	0.00	0.00	0.00	0.00	0.00	0.00	0.13	0.00	0.00
1H	2.6	0.04	0.00	0.01	0.03	0.02	0.00	0.01	0.10	0.00	0.01
1H	7.4	0.00	0.00	0.01	0.33	0.00	0.00	0.00	0.09	0.02	0.00
1H	10.5	0.00	0.00	0.51	0.02	0.00	0.00	0.00	0.01	0.00	0.00
1H13C	2.6	0.02	0.02	0.00	0.03	0.01	0.01	0.00	0.03	0.00	0.01
1H13C	7.4	0.00	0.00	0.00	0.00	0.00	0.01	0.00	0.19	0.00	0.00
1H13C	10.5	0.00	0.00	0.00	0.01	0.01	0.00	0.00	0.00	0.00	0.00
1H+13C	2.6	0.01	0.01	0.01	0.09	0.01	0.02	0.00	0.09	0.01	0.01
1H+13C	7.4	0.00	0.00	0.02	0.01	0.00	0.01	0.00	0.02	0.00	0.00
1H+13C	10.5	0.00	0.00	0.01	0.00	0.00	0.00	0.00	0.00	0.00	0.00
13C1H	2.6	0.00	0.00	0.04	0.00	0.00	0.04	0.01	0.11	0.05	0.00
13C1H	7.4	0.01	0.00	0.07	0.01	0.01	0.00	0.00	0.00	0.00	0.01
13C1H	10.5	0.00	0.00	0.02	0.07	0.00	0.01	0.00	0.21	0.06	0.02
FP	2.6	0.00	0.00	0.00	0.01	0.01	0.01	0.00	0.00	0.01	0.00
FP	7.4	0.00	0.00	0.12	0.00	0.00	0.00	0.00	0.01	0.00	0.00
FP	10.5	0.00	0.00	0.01	0.08	0.00	0.01	0.00	0.00	0.00	0.00

Table S11a. Hyperparameter importance for MLP Dual-Stream models

Data across different pH values. The table presents only the hyperparameters with relative importance ≥ 0.01, as determined by Optuna's *get_param_importances()* method. For each pH condition, the most impactful parameters – such as optimizer, learning rate, and dropout mechanisms – are reported. Less influential hyperparameters were omitted for clarity.

ML architecture	optimizer	lr	batch_size	use_cross_attention	mlp_num_layers	final_hidden_dim	c_h_l0
MLP Dual-Stream pH 2.6	0.814	0.089	0.054	0.024	0.011		
MLP Dual-Stream pH 7.4	0.612	0.084	0.108		0.178		
MLP Dual-Stream pH 10.5	0.055	0.116		0.732	0.031	0.025	0.014

Table S11b. Hyperparameter importance for CNN Dual-Stream models

Data across different pH values. Reported values reflect only the hyperparameters with measurable impact (importance ≥ 0.01). The most prominent contributors include learning rate, global dropout, and batch normalization. Architectural features such as convolutional depth or kernel configurations had lower importance and were excluded from the summary.

ML architecture	dropout	optimizer	use_bn	lr	n_conv_h	n_conv_c	batch_size	h_ch_l0	h_k_l0	c_k_l0	c_s_l0	h_s_l0	embed_dim	fc_hidden
CNN Dual-Stream pH 2.6	0.398	0.166	0.129	0.098	0.089	0.043	0.013	0.012	0.011	0.010				
CNN Dual-Stream pH 7.4	0.171	0.055	0.041	0.493	0.016	0.061		0.035			0.057	0.026	0.019	
CNN Dual-Stream pH 10.5	0.104	0.015	0.242	0.366	0.016	0.105		0.03		0.016			0.020	0.067

Table S11c. Hyperparameter importance for 2D CNN models

Models trained on stacked ^{1}H+^{13}C spectra. This table includes only parameters with importance ≥ 0.01. Dominant factors include learning rate and dropout, especially in acidic and neutral pH environments. Several convolutional-layer-related hyperparameters are present, but their influence is limited compared to optimization and regularization settings.

ML architecture	optimizer	cnn_kernel_size	lr	cnn_dropout	final_dropout	cnn_linear_out	cnn_batch_norm	cnn_out_channels_l1	final_hidden_dim	cnn_num_layers	cnn_out_channels_l0	batch_size
2D CNN Stacked pH 2.6	0.357	0.245	0.115	0.098	0.091	0.023	0.018	0.015	0.015	0.012		
2D CNN Stacked pH 7.4	0.013		0.562	0.288					0.078		0.043	
2D CNN Stacked pH 10.5	0.030	0.010	0.465	0.068		0.011	0.068		0.045	0.010	0.018	0.272

Table S12. Overview of all optimized Hyperparameters. All hyperparameters were tuned with Optuna across different model architectures. The table includes their respective search ranges.

Model Architecture	Hyperparameter	Search Range
SVR	C	1e−5 to 100 (log-scale)
	epsilon	1e−7 to 1.0 (log-scale)
	tol	1e−5 to 1e−1 (log-scale)
	gamma	1e−5 to 100 or ['scale', 'auto']
XGBoost	max_depth	1 to 20
	learning_rate	1e−5 to 0.5 (log-scale)
	subsample	0.1 to 1.0
	colsample_bytree	0.1 to 1.0
	colsample_bylevel	0.1 to 1.0
	colsample_bynode	0.1 to 1.0
	min_child_weight	0.1 to 10.0
	gamma	0.0 to 5.0
	reg_alpha	1e−8 to 100 (log-scale)
	reg_lambda	1e−8 to 100 (log-scale)
	max_delta_step	0.0 to 10.0
	grow_policy	['depthwise', 'lossguide']
MLP (incl. Dual & CA)	mlp_num_layers	1 to 6
	mlp_dropout	0.0 to 0.6 (step 0.1)
	units per layer	32 to 1024 (log-scale)
	embed_dim	16 to 512 (log-scale)
	final_hidden_dim	32 to 512 (log-scale)
	final_dropout	0.0 to 0.6
	optimizer	['Adam', 'SGD', 'RMSProp']
	learning_rate	1e−5 to 1e−3 (log-scale)
	batch_size	16 to 256 (log-scale)
	use_cross_attention	True / False
	ca_heads	1, 2, 4, or 8
CNN (incl. 1D, 2D, Dual)	num_conv_layers	1 to 3
	num_filters	16 to 512 (log-scale)
	kernel_size	3 to 9
	stride	1 or 2
	fc_units_l0 / l1	32 to 1024 (log-scale)
	dropout_rate	0.0 to 0.6
	use_batch_norm	True / False
	optimizer	['Adam', 'SGD', 'RMSProp']
	learning_rate	1e−5 to 1e−3 (log-scale)
	batch_size	16 to 256 (log-scale)
	use_cross_attention	True / False (Dual only)
	ca_heads	1, 2, 4, or 8 (Dual only)

REFERENCES

(1) A. Leniak. *https://github.com/Prospero1988/Demiurge*. Github.

(2) Rogers, D.; Hahn, M. Extended-Connectivity Fingerprints. *J Chem Inf Model* 2010, *50* (5), 742–754. https://doi.org/10.1021/ci100050t.

(3) RDKit: Open-Source Cheminformatics. https://www.rdkit.org (accessed 2024-10-14).

(4) Steinbeck, C.; Krause, S.; Kuhn, S. NMRShiftDBConstructing a Free Chemical Information System with Open-Source Components. *J Chem Inf Comput Sci* 2003, *43* (6), 1733–1739. https://doi.org/10.1021/ci0341363.

(5) Steinbeck, C.; Kuhn, S. NMRShiftDB – Compound Identification and Structure Elucidation Support through a Free Community-Built Web Database. *Phytochemistry* 2004, *65* (19), 2711–2717. https://doi.org/10.1016/j.phytochem.2004.08.027.

(6) Kuhn, S.; Schlörer, N. E. Facilitating Quality Control for Spectra Assignments of Small Organic Molecules: Nmrshiftdb2 – a Free In-house NMR Database with Integrated LIMS for Academic Service Laboratories. *Magnetic Resonance in Chemistry* 2015, *53* (8), 582–589. https://doi.org/10.1002/mrc.4263.

(7) Bremser, W. Hose — a Novel Substructure Code. *Anal Chim Acta* 1978, *103* (4), 355–365. https://doi.org/10.1016/S0003-2670(01)83100-7.

(8) *NMRshiftDB2 Predictors*. https://sourceforge.net/p/nmrshiftdb2/wiki/PredictorJars/ (accessed 2024-10-14).

(9) Üstün, B.; Melssen, W. J.; Buydens, L. M. C. Visualisation and Interpretation of Support Vector Regression Models. *Anal Chim Acta* 2007, *595* (1–2), 299–309. https://doi.org/10.1016/j.aca.2007.03.023.

(10) Chen, T.; Guestrin, C. XGBoost. In *Proceedings of the 22nd ACM SIGKDD International Conference on Knowledge Discovery and Data Mining*; ACM: New York, NY, USA, 2016; pp 785–794. https://doi.org/10.1145/2939672.2939785.

(11) Bentéjac, C.; Csörgő, A.; Martínez-Muñoz, G. A Comparative Analysis of Gradient Boosting Algorithms. *Artif Intell Rev* 2021, *54* (3), 1937–1967. https://doi.org/10.1007/s10462-020-09896-5.
